# Engineering Ru@Ir Core–Shell Nanoparticles on Titanium Oxynitride–Graphene Support for a Highly Active and Durable pH-Universal Hydrogen Evolution Reaction

**DOI:** 10.1021/acscatal.5c02831

**Published:** 2025-07-21

**Authors:** A. Popović, I. Marić, M. Bele, E. Rems, M. Huš, L. Pavko, F. Ruiz-Zepeda, L. Bijelić, B. Grgur, N. Hodnik, M. Smiljanić

**Affiliations:** † Department of Materials Chemistry, National Institute of Chemistry, Hajdrihova 19, 1000 Ljubljana, Slovenia; ‡ Faculty of Technology and Metallurgy. University of Belgrade, Karnegijeva 4, 11000 Belgrade, Serbia; § Radiation Chemistry and Dosimetry Laboratory, Division of Materials Chemistry, Ruđer Bošković Institute, Bijenička 54, 10000 Zagreb, Croatia; ∥ Faculty of Chemistry and Chemical Technology, University of Ljubljana, Večna pot 113, 1000 Ljubljana, Slovenia; ⊥ Department of Catalysis and Chemical Reaction Engineering, National Institute of Chemistry, Hajdrihova 19, 1000 Ljubljana, Slovenia; # Association for Technical Culture of Slovenia (ZOTKS), Zaloška 65, 1000 Ljubljana, Slovenia; ∇ Institute for the Protection of Cultural Heritage of Slovenia (ZVKDS), Poljanska 40, 1000 Ljubljana, Slovenia; ○ Department of Physics and Chemistry of Materials, Institute of Metals and Technology, Lepi pot 11, 1000 Ljubljana, Slovenia; ◆ Jožef Stefan International Postgraduate School, Jamova cesta 39, 1000 Ljubljana, Slovenia; ¶ University of Nova Gorica, Vipavska 13, 5000 Nova Gorica, Slovenia

**Keywords:** hydrogen evolution reaction, iridium, ruthenium, core−shell structures, TiO_*x*_N_*y*_, MSI, DFT

## Abstract

The rational design of electrocatalysts with high activity, durability, and low precious metal content is key to advancing hydrogen production via water electrolysis. Here, we present a multifunctional electrocatalyst based on Ru@Ir core–shell nanoparticles anchored on a conductive titanium oxynitride–graphene hybrid support (Ru@Ir/TiO_*x*_N_*y*_-C), achieving superior performance for the hydrogen evolution reaction (HER) in both acidic and alkaline media. The combination of the core–shell Ru@Ir architecture and the strong metal–support interaction (MSI) with TiO_*x*_N_*y*_ optimizes hydrogen and hydroxide adsorption energies, as confirmed by X-ray photoelectron spectroscopy and density functional theory (DFT) calculations. In alkaline media, Ru@Ir/TiO_*x*_N_*y*_-C outperforms commercial Pt/C with a remarkably low overpotential of 13 mV at 10 mA cm^–2^ and high mass activity, while in acidic conditions, it rivals Pt/C and surpasses monometallic analogs. The long-term stability of the composite is attributed to the enhanced nanoparticle anchoring and structural integrity provided by the TiO_*x*_N_*y*_ support. This work shows that combining core–shell nanostructures with engineered conductive supports can effectively replace platinum in HER applications. Such a nanocomposite strategy offers a versatile platform for the development of advanced electrocatalysts across a broad range of energy conversion reactions.

## Graphical abstract

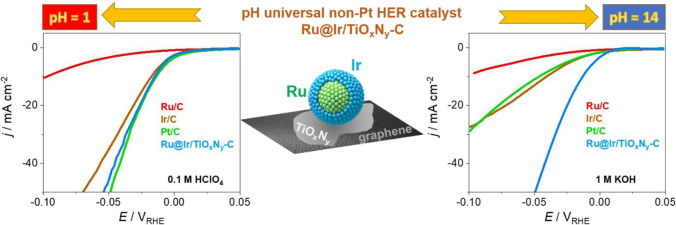

## 1 Introduction

Electrochemical energy conversion devices such as electrolyzers and fuel cells will play a crucial role in the advancements of hydrogen energy and clean chemical production.^1^ Water electrolysis (WE) is expected to become the leading technology for the decentralized production of green hydrogen in the coming years.^2^ In WE, hydrogen is produced by an electrochemical hydrogen evolution reaction (HER), while the counter-process is the oxygen evolution reaction (OER). The efficiency and feasibility of WEs depend largely on the electrocatalysts used to facilitate water splitting. Therefore, new materials with improved electrocatalytic behavior for HER (and OER) are constantly being sought using different strategies, such as nanostructuring, surface functionalization, heteroatom doping, compositional engineering, single-atom catalysis, and conductive supports.^3^ These experimental approaches can be easily complemented by the increasing application of computational methods^4^ and machine learning.^5^

Combining two (or more) metals is a classical and very effective method to obtain materials with improved electrocatalytic properties.^6–8^ In multimetallic nanocomposites, new active sites with unprecedented activity compared to single metal catalysts can appear due to electronic and/or geometric effects. The electronic effect arises from the interaction between different metals in close contact, which changes the electronic density of the active sites by electron transfer or a shift in electronic levels, including the d-band center.^4,9^ The geometric effect refers to the distorted arrangement and distribution of atoms within the crystal lattice, which has a similar influence on the electrocatalytic activity as the electronic effect. Since platinum (Pt) is the benchmark catalyst for HER,^10^ the goal is always to obtain materials that are equivalent or ideally better than Pt. Ruthenium (Ru) has recently gained increasing attention in HER catalysis,^11,12^ due to its moderate activity, good electrochemical stability, and significantly lower price than Pt. Various Ru nanostructures have been explored in the literature and have been shown to perform comparably to Pt/C benchmarks, especially in alkaline electrolytes.^11,13,14^ This has naturally sparked interest in Ru-based bimetallic materials. For instance, the Ru–Pt catalyst showed excellent HER activity throughout the entire pH spectrum, which can be attributed to the simultaneous optimization of water dissociation and hydrogen-binding energy.^15^ Epitaxial growth of unconventional fcc-Ru-nanorods over Au-fcc nanowires has been reported to be a viable strategy to obtain high-performance HER catalysts.^16^ Mesoporous Pd–Ru core–shell nanorods exhibited excellent HER performance due to the optimized water activation properties.^17^ In another study, Ru–Rh nanorings were featured with exceptional HER activity and robust operation in the whole pH range due to the simultaneous tuning of hydrogen adsorption energy and water dissociation barrier.^18^ A RuIr nanoalloy on nitrogen-enriched carbon support showed excellent HER activity in both acidic and alkaline media.^19^ Similarly, there are reports of highly active Ru-alloys with transition metals (TMs),^20–22^ albeit mainly in alkaline electrolytes.

Another way to fine-tune the active sites for HER is to use metal–support interaction (MSI), a very popular concept in catalysis.^23^ MSI is based on the use of metal oxides, which can tune the reactivity of supported active sites via electronic and geometric effects (i.e., in a similar way as bimetallics).^24–26^ MSI can also involve partial encapsulation of active sites with a thin layer of oxide support^27,28^ and enhanced anchoring of the nanoparticles with support,^29^ which can alter both the activity and stability of catalytic composites. TiO_2_ is one of the most commonly used support materials in heterogeneous catalysis due to its low cost, low toxicity, distinct electronic properties, and high corrosion resistance. However, in electrocatalysis, the support materials must have a high surface area and electrical conductivity, which can limit the application of TiO_2_. In some cases, blending TiO_2_ with carbon^30^ or adding higher amounts of active compounds^31^ can alleviate these issues. For instance, Umicore’s benchmark OER catalyst is supplied with 75 wt % IrO_2_ on TiO_2_. However, such high metal utilization is not desirable, especially in the case of scarce and expensive precious metals. Our group has addressed these limitations by partially nitriding TiO_2_ to form conductive titanium oxynitride (TiO_*x*_N_*y*_),^32^ which can be mixed with various carbon supports to further increase both surface area and conductivity.^33^ The application of TiO_*x*_N_*y*_ as a support for Ir nanoparticles was shown to be highly beneficial for OER catalysis,^32–34^ which was linked with the MSI between TiO_*x*_N_*y*_ and Ir.^35^ Furthermore, we combined TiO_*x*_N_*y*_ with Pt to obtain highly performing catalysts for HER^29^ and the oxygen reduction reaction.^36^ In the case of HER, MSI resulted in a slight weakening of the binding of chemisorbed H intermediates on Pt sites supported on TiO_*x*_N_*y*_ compared to Pt/C, which is the main parameter affecting HER activity in acidic electrolytes. At the same time, the durability of Pt/TiO_*x*_N_*y*_ was improved as Pt was grafted more strongly on TiO_*x*_N_*y*_ than on carbon, which prevented degradation mechanisms during HER, such as particle migration, coalescence, or detachment. In our recent work, we combined low-loaded Ru active sites (6 wt %) with TiO_*x*_N_*y*_-carbon support and explored HER on the novel Ru/TiO_*x*_N_*y*_-C composite.^37^ Versatile effects of MSI on supported Ru particles led to the exceptional performance toward alkaline HER, as both H adsorption energy and water dissociation barrier were favorably affected. The effect of MSI between TiO_*x*_N_*y*_ and low-loaded Ru sites had a much higher influence on HER activity than in the case of Pt. Therefore, besides its exceptional alkaline HER performance, the Ru/TiO_*x*_N_*y*_-C composite could also be considered as a platform for further material engineering to obtain highly active pH-universal HER catalysts.

In the present work, Ru@Ir core–shell nanoparticles supported on a TiO_*x*_N_*y*_-graphene composite (hereafter referred to as Ru@Ir/TiO_*x*_N_*y*_-C) were nanoengineered and explored as HER electrocatalysts. The choice of these two metals was guided by the fact that they are both active for HER, susceptible to performance enhancement with MSI when interfaced with TiO_*x*_N_*y*_, and that many previous reports showed benefits of combining Ru and Ir in various nanostructures on HER reactivity.^19,38–41^ Moreover, our previous work showed the distinct effect of MSI induced by TiO_*x*_N_*y*_ on the catalytic behavior of both Ru (for HER) and Ir (for OER), making them the perfect combination to study the cumulative effect of functionalized nanostructures and MSI. Structural characterization of the material was performed using X-ray diffraction (XRD), X-ray photoelectron spectroscopy (XPS), and transmission electron microscopy (TEM). Ru@Ir/TiO_*x*_N_*y*_-C exhibited excellent HER activity, which was significantly higher than that of the Pt/C benchmark in alkaline media and on par with it in acidic media. In addition, Ru@Ir/TiO_*x*_N_*y*_-C provided stable HER operation as shown by electrochemical and identical location TEM (IL-TEM) studies. Density functional theory (DFT) calculations revealed that the adsorption of both hydrogen atoms and hydroxide species was favorably tuned thanks to MSI and core-shell structural effects.

## 2 Experimental Section

### 2.1 Synthesis and Characterization of Ru@Ir/TiO_*x*_N_*y*_-C Composite

Graphene oxide (GO) was prepared by a modified Hummers method as previously documented.^42^ In a 5-L beaker, a mixture of 1000 mL of 96 wt % sulfuric acid and 110 mL of 85 wt % phosphoric acid was prepared. Graphite (Imerys) was introduced and KMnO_4_ was added incrementally every 24 h until a total amount of 5 wt.-eq was reached. After 2 days of continuous stirring, the reaction was quenched with ice, followed by the addition of 30 wt % H_2_O_2_ until the color changed from purple to yellow. The resulting GO settled overnight and the supernatant was replaced with ultrapure water. Further purification steps included dispersion in 5 wt % HCl, centrifugation, and redispersion in ultrapure water in several cycles. The final GO suspension was treated in an ice bath with a homogenizer for 1 h to exfoliate the product.

To prepare the amorphous TiO_2_ coating, 2 g of graphene oxide was added to 0.02 mol of titanium isopropoxide (Sigma-Aldrich, 97%) dissolved in 0.24 mol of 2-propanol (Honeywell, puriss, p.a.). The slurry was thoroughly mixed in a mortar and 0.4 mol (7.2 mL) ultrapure water (resistivity 18.2 MΩ cm, Milli-Q) was added to the mixture to hydrolyze the titanium isopropoxide. The sample was dried in a freeze-dryer to completely remove the remaining water. In the next step, Ru was deposited on the obtained TiO_2_/GO composite. For this purpose, 0.16 mmol RuCl_3_·*x*H_2_O (Apollo Scientific) was dissolved in 1.6 mL acetone and the solution was added to 0.3 g amorphous TiO_2_/GO composite. The resulting slurry was mixed in a mortar until the acetone was completely evaporated. The dried powder was then placed in a crucible and annealed in NH_3_ atmosphere (NH_3_ flow rate of 100 mL min^–1^). In the first step, the temperature was increased to 250 °C min^–1^ for 2 h at a rate of 1 °C min^–1^. Subsequently, the temperature was increased to 750 °C min^–1^ at a rate of 10 °C min^–1^ for 9 h and then cooled to room temperature at a rate of 10 °C min^–1^. Finally, to deposit Ir, 0.17 mmol IrBr_3_·*x*H_2_O (AlfaAesar) was dissolved in 0.7 mL water at 80 °C. Similar to the previous step, this solution was added to the Ru/TiO_*x*_N_*y*_-C powder and this slurry was mixed in a mortar while hot air was simultaneously used to dry the slurry. The slurry was then dried for an additional 1 h at 50 °C in an oven. Once the slurry was completely dried, the sample was placed in a crucible and annealed in NH_3_ atmosphere (NH_3_ flow rate of 100 mL min^–1^). In the first step, the temperature was increased to 120 °C min^–1^ for 2 h at a rate of 7 °C min^–1^. Subsequently, the temperature was increased to 450 °C min^–1^ at a rate of 2 °C min^–1^ for 1 h and then cooled to room temperature at a rate of 3 °C min^–1^ to obtain the final Ru@Ir/TiO_*x*_N_*y*_-C product. For the preparation of the Ru@Ir/C sample, Vulcan XC72 was used as a support, while the procedure for the deposition of Ru and Ir was the same as for the Ru@Ir/TiO_*x*_N_*y*_-C sample. The final loading of the metals in all catalysts was determined by inductively coupled plasma-optical emission spectroscopy (ICP-OES).^43^ In the case of Ru@Ir/C loadings of Ru and Ir were 3.6 and 7.9 wt %, respectively. The content of Ru and Ir in the Ru@Ir/TiO_*x*_N_*y*_-C sample was evaluated at 5.2 and 8.8 wt %, respectively. For comparison of the HER electrocatalytic properties, a homemade Ru/C catalyst with 10 wt % Ru was used, while Ir/C and Pt/C benchmarks were purchased from Premetek (both with 20 wt %).

The Ru@Ir/TiO_*x*_N_*y*_-C catalyst was further characterized by X-ray diffraction (XRD), aberration-corrected scanning transmission electron microscopy (AC-STEM), and X-ray photoelectron spectroscopy (XPS). X-ray diffractograms were obtained using a D4 Endeavor and a Bruker AXS diffractometer with Cu–Kα radiation and a Sol-X energy dispersion detector. For a detailed microstructural study, a Cs probe-corrected scanning transmission electron microscope (Jeol ARM 200 CF) at 80 kV was used. XPS analyses were performed with a Versa probe 3 AD device (Phi, Chanhassen, MN, US) using a monochromatic Al Kα X-ray source at 15 kV and 3.3 mA emission current. The powder samples mounted on double-sided adhesive tape were analyzed on a 1 × 1 mm^2^ spot with the charge neutralizer active throughout the measurements. High-resolution (HR) XPS spectra were acquired with a pass energy of 27 eV and a binding energy (BE) step of 0.1 eV, while survey spectra were acquired with a pass energy of 224 eV and a BE step of 0.8 eV. To improve the signal-to-noise ratio, each spectrum underwent at least 20 sweeps. The binding energy scale of the XPS spectra was calibrated using the C 1s peak at a BE of 284.4 eV, which corresponds to the partially graphitized carbon support (part) in the samples. Data processing, including fitting, was carried out using MultiPak 9.0 software, and the Shirley background subtraction method was consistently applied to all measurements.

In the Ru 3d region, four main doublet peaks corresponding to Ru^0^, RuO_2_, RuO_3,_ and RuO_4_ were identified for both the Ru/C and Ru@Ir/TiO_*x*_N_*y*_-C samples. The spin–orbit splitting value was kept constant at 4.17 eV for all bands between Ru 3d5/2 and Ru 3d3/2. The peak shape was chosen to be asymmetric for Ru0 and symmetric (80% Gaussian, 20% Lorentz) for the oxide species. The full width at half-maximum (fwhm) for both samples was set to 0.7 for Ru^0^, 1.1 for RuO_2_, 1.4 for RuO_2_, and 1.5 for RuO_4_.

### 2.2 Electrochemical Characterization and HER Investigations

In all cases, the catalyst inks were prepared by mixing the catalyst powder with ultrapure water (18.2 MΩ cm) at a ratio of 1 mg/mL in an ice-cooled ultrasonic bath to ensure complete dispersion. Glassy carbon rotating disk electrodes (RDE) were used as substrates for the deposition of the catalyst thin films and cleaned by hand-polishing with alumina slurry. Catalyst ink (20 μL) was pipetted onto the RDEs (0.196 cm^2^, Pine) and dried in a closed desiccator. Finally, the dried films were covered with 5 μL of Nafion (Sigma, 5% solution in a mixture of lower aliphatic alcohols and water) diluted in isopropanol (1/50 v/v).

Initial HER activity/stability studies were performed in Ar-saturated alkaline or acidic electrolytes in a thin-film rotating disk electrode (TF-RDE) setup. In the case of the alkaline media (1 M KOH, Merck), the experiments were carried out in a Teflon cell, while a glass cell was used for the acidic electrolyte (0.1 M HClO_4_, Carl Roth). In all cases, a reversible hydrogen electrode (Hydroflex from Gaskatel) and a glassy carbon rod were used as reference and counter electrodes, respectively. The catalyst films were activated by cyclic voltammetry (300 mV/s, 50 cycles, 0.05–1 V_RHE_, followed by three cycles in the same potential window at a scan rate of 50 mV/s) to achieve a stable and reproducible electrochemical response. The HER activities were measured by recording the polarization curves at a scan rate of 10 mV/s in the potential range of 0.1 to −0.2 V_RHE_ in alkaline or 0.1 to −0.1 V_RHE_ in acidic electrolyte. The stability of the Ru@Ir/TiO_x_N_y_-C and Ru@Ir/C counterparts was tested by extensive potential cycling (10,000 cycles, 100 mV/s, potential window between 0.1 and −0.1 V_RHE_) in the TF-RDE setup. Additional information on the stability of Ru@Ir/TiO_*x*_N_*y*_-C was obtained using STEM at an identical location in conjunction with the modified floating electrode (MFE).^44^ In this case, a gold TEM grid was coated with 5 μL of the catalyst ink and the excess liquid was removed to ensure the formation of a very thin catalyst layer, allowing for easier IL-STEM imaging. The untreated catalyst was imaged at several locations before the grid was mounted in MFE and subjected to a potentiostatic durability test at an overvoltage of 50 mV_RHE_ for 4 h in 1 M KOH electrolyte. IL-STEM Annular Dark Field (ADF) imaging was then performed at the same locations to observe possible changes in the nanostructure of the material.

### 2.3 Density Functional Theory Calculations

Density functional theory (DFT) modeling was performed using the projector augmented-wave method (PAW)^45^ implemented in the Vienna Ab initio Simulation Package (VASP).^46^ The Perdew–Burke–Ernzerhof (PBE) generalized-gradient approximation (GGA) functional was employed^47^ with PAW pseudopotentials. Spin polarization was considered for isolated atoms and the hydroxyl radical. Nonspherical contributions related to the density gradient in PAW spheres were included. The dispersion interaction was accounted for according to the DFT-D3 method of Grimme et al.^48^ The energy cutoff for the plane-wave basis set was set as 520 eV. The width of the Gaussian smearing was 0.05 eV. The Brillouin zone was sampled based on a Γ-centered mesh with a reciprocal space resolution of 2π·0.04 Å^–1^. Structural optimizations were performed until the forces dropped below 0.01 eV Å^–1^. In optimizations of catalyst surface slabs, the two top layers were fully relaxed, and the two bottom layers were fixed in their bulk positions. Periodic interactions between surface slabs were prevented through approximately 15 Å thick vacuum layers. Energy corrections to the Gibbs free energy were evaluated in the harmonic approximation, as implemented in VASPKIT.^49^ Corrections for isolated species include zero-point energy (ZPE) and thermal vibrational, translational, electronic, and rotational contributions, whereas only adsorbent vibrations were considered for adsorbent–adsorbate complexes. Adsorbent vibrations were assumed to be independent of the surface coverage and were evaluated in the limit of low coverage (one adsorbate in a supercell, corresponding to a 1/16 monolayer coverage) and considered additive. Phonon calculations were performed using the finite difference method, with the width of displacement for each ion set to 0.015 Å. Vibrational frequencies below 50 cm^–1^ were raised to 50 cm^–1^. Structures were visualized by VESTA^50^ and Ovito.^51^

## 3 Results and Discussion

### 3.1 Characterization of Ru@Ir/TiO_*x*_N_*y*_-C Catalyst

The characterization of the Ru@Ir/TiO_*x*_N_*y*_-C and carbon-supported catalysts involved the application of various techniques to gain a comprehensive insight into their properties. A comparison of the XRD patterns of Ru/C, Ir/C, Ru@Ir/C, and Ru@Ir/TiO_*x*_N_*y*_-C is shown in Figure 1, together with STEM and EDS analysis of the Ru@Ir/TiO_*x*_N_*y*_-C composite.

**Figure 1:**
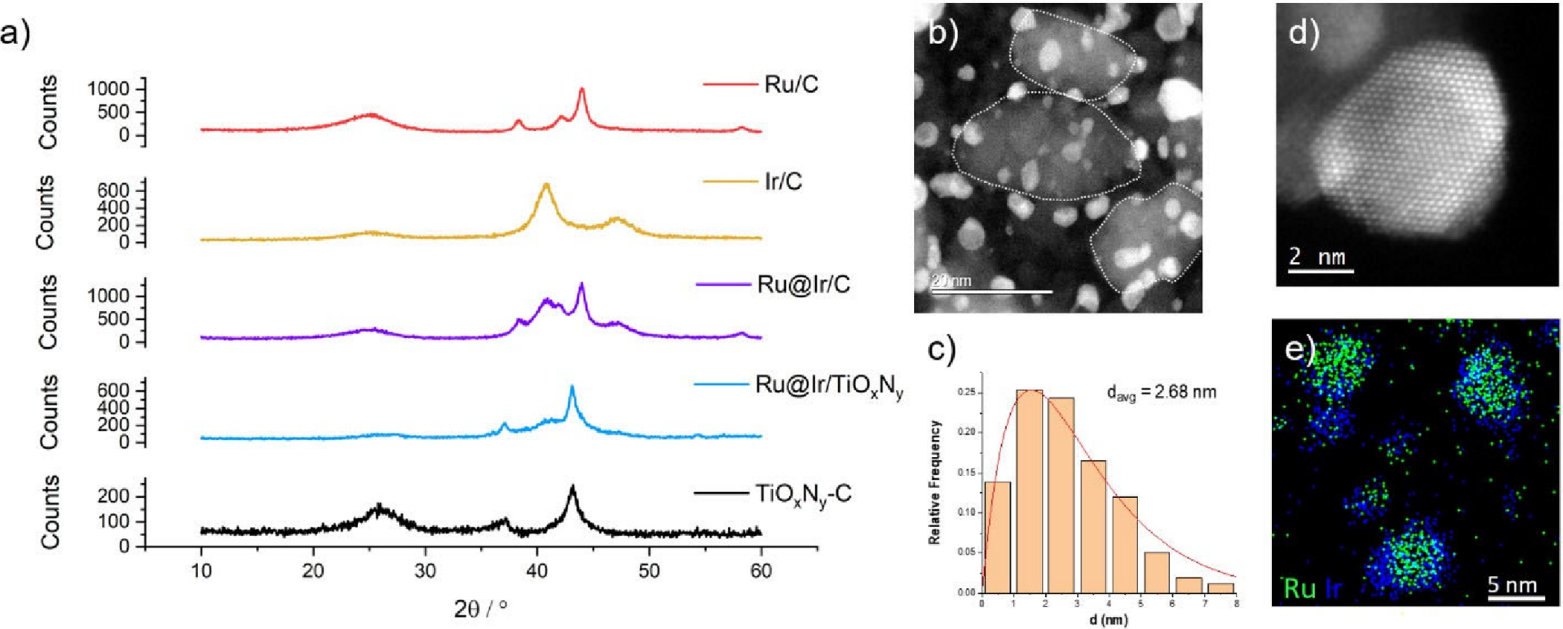
(a) XRD patterns of the investigated electrocatalytic composites; (b) STEM ADF imaging of Ru@Ir/TiO_*x*_N_*y*_-C sample and (c) corresponding particle size distribution; (d) high-resolution STEM ADF image of a core–shell Ru@Ir nanoparticle; and (e) EDS mapping of Ru@Ir nanoparticles (green stands for the Ru core, blue represents the Ir shell).

Ru/C and Ir/C samples show characteristic reflections of metallic hcp-Ru (00-001-1253) and fcc-Ir (00-001-1216) phases, respectively. Support TiO_*x*_N_*y*_-C shows two peaks at 37° and 43° related to cubic TiON (00-049-1325) and a broad peak at 26° originating from the graphene template. It can be seen that the Ru@Ir/C material shows characteristic reflections corresponding to separated metallic Ir and Ru phases. STEM ADF images and EDS analyses of the Ru@Ir/C sample are shown in Figure S1. They show that core–shell nanostructures with an average particle size of about 3.2 nm were formed, although larger particles with diameters between 5 and 10 nm (and even larger) were also detected. Elemental mapping shows that the NPs consist of a separated Ir shell and a Ru core, implying that the initially deposited Ru NPs acted as a seed for the subsequent growth of the Ir shell. In the case of Ru@Ir/TiO_*x*_N_*y*_-C, XRD analysis is complicated by the masking of some reflections by the dominant TiO_*x*_N_*y*_ peaks at 37° and 43° (00-049-1325). The only recognizable feature of the Ru@Ir particles is a broad peak between 39.4° and 42.4°, which is also present in the spectrum of Ru@Ir/C and corresponds to the diffractions of Ru 002 (42.4°) and Ir 111 (41°). The appearance of two new peaks (at 27.4° and 54.2°) may be related to the XRD reflections of rutile IrO_*x*_. To further analyze the Ru@Ir/TiO_*x*_N_*y*_-C sample, STEM ADF imaging was performed. The corresponding particle size distribution and the elemental map are shown in the right panel of Figure 1. The nanoparticles are well distributed over the TiO_*x*_N_*y*_-C support and mainly adhere to the TiO_*x*_N_*y*_ flakes, which are marked by dotted white lines in Figure 1(b) for better visualization. The particle size distribution shows that the average diameter of Ru@Ir particles is around 2.68 nm (Figure 1(c)). A core–shell structure of the Ru@Ir nanoparticles is evidenced in Figure 1(d) by the difference in Z contrast when imaging Ru (*Z* = 44) and Ir (*Z* = 77). More examples are given in Figures S2 and S3. In addition, elemental mapping of Ru@Ir (Figures 1(e) and S4) confirms the formation of core–shell nanoparticles grafted onto a TiO_*x*_N_*y*_-C support. In some cases, the formation of small monometallic Ir nanoparticles (diameter less than 1.5 nm) was observed in the Ru@Ir/TiO_*x*_N_*y*_-C sample (Figures 1(e) and S4).

X-ray photoelectron spectroscopy was systematically performed for all compared samples (Ru/C, Ir/C, Ru@Ir/C, and Ru@Ir/TiO_*x*_N_*y*_-C) to obtain information on their chemical composition and to gain insights into possible MSI effects. The survey spectra confirmed the presence of the corresponding elements, namely Ru, Ir, C, N, O, and Ti, in the respective samples (Figure S5). To gain deeper insight into MSI, high-resolution (HR) spectra of Ru 3d were investigated in detail in Figure 2 with respect to the C 1s and C=C binding energies (BE). A similar analysis was performed for Ir 4f and presented in Figure 3. In the case of Ru/C, the metallic Ru^0^ and Ru oxide species (RuO_*x*_) were identified after peak fitting of the HR Ru 3d spectra (Figure 2a) according to the parameters described in the Section 2. Also in the case of Ir/C, the peak fitting of the Ir 4f HR spectra showed the presence of metallic Ir and IrO_2_ (Figure 3a).

**Figure 2:**
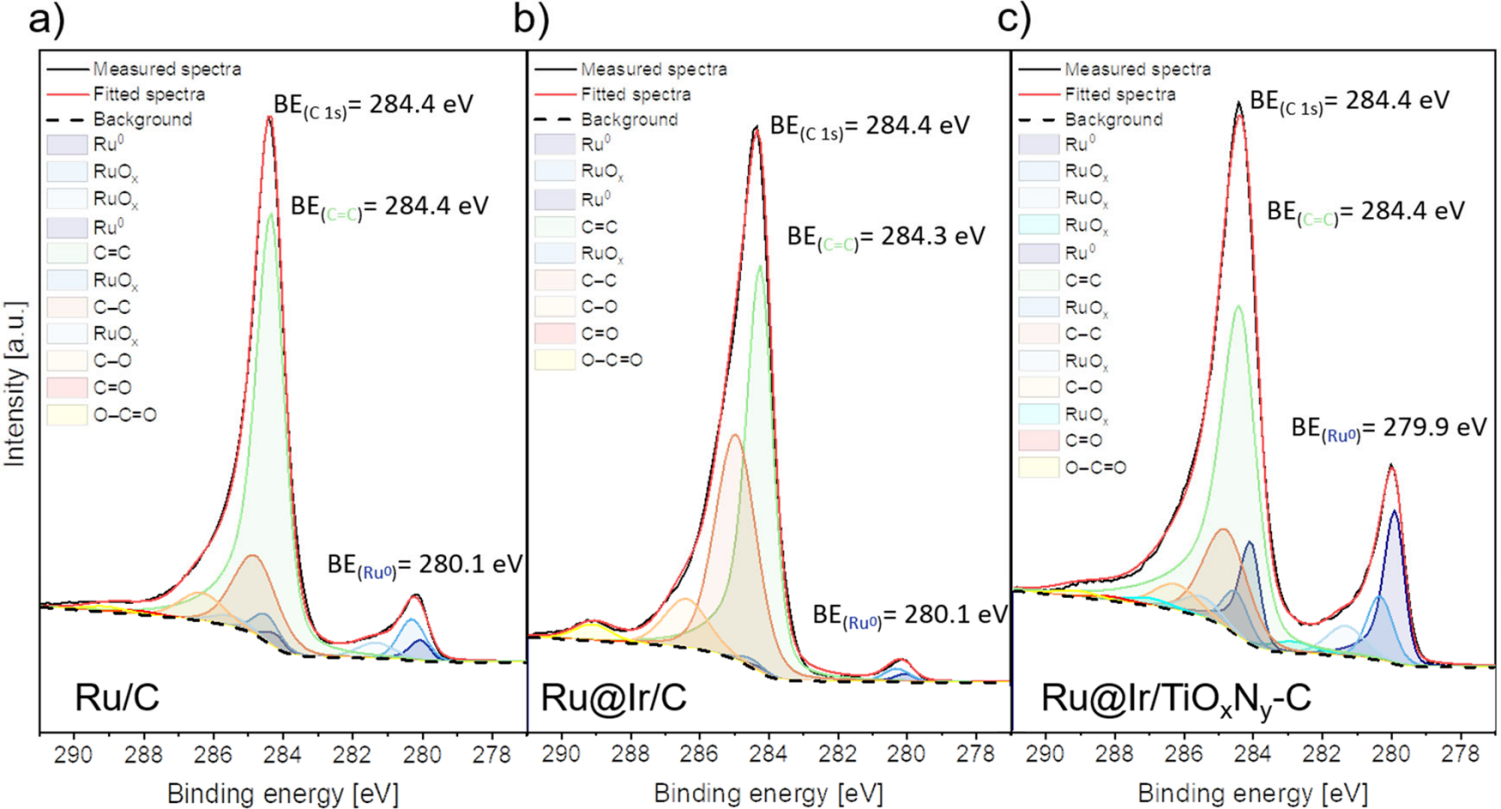
XPS characterization of the Ru 3d region for samples: (a) Ru/C, (b) Ru@Ir/C, and (c) Ru@Ir/TiO_*x*_N_*y*_-C.

**Figure 3:**
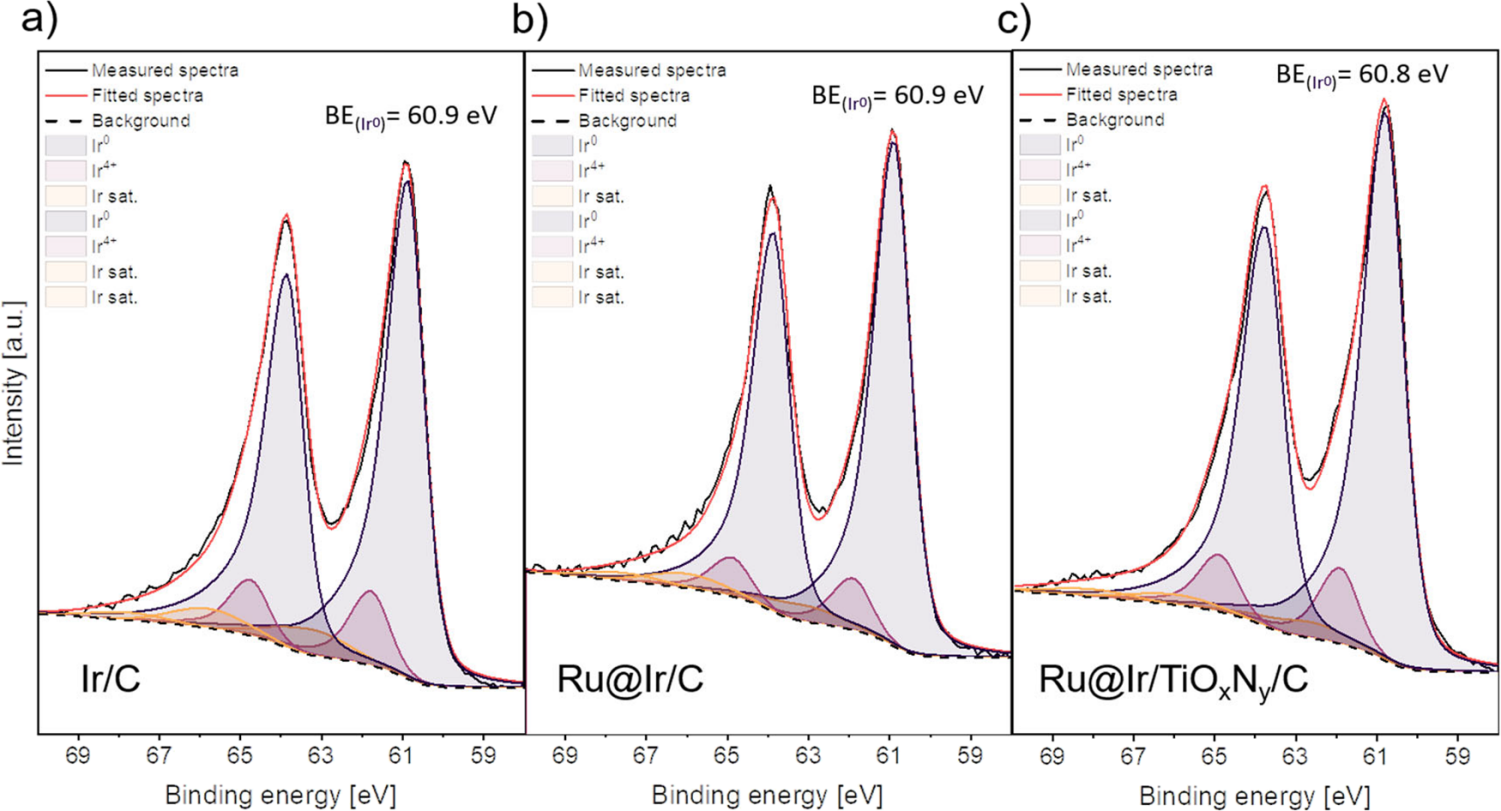
XPS characterization of the Ir 4f region for samples: (a) Ir/C, (b) Ru@Ir/C, and (c) Ru@Ir/TiO_*x*_N_*y*_-C.

For the Ru@Ir/C sample (Figure 2b), the same Ru species were identified in the Ru 3d HR spectra as in the Ru/C benchmark at the same BE values. Similarly, the same Ir species as in Ir/C were found in the spectra of Ru@Ir/C (Figure 3b) and are placed at the same BEs. In the case of the Ru@Ir/TiO_*x*_N_*y*_-C sample (Figure 2c), metallic Ru and Ru oxide species were detected (with the additional RuO_*x*_ peak present toward higher BE in contrast to the Ru@Ir/C sample). Compared to the Ru/C and Ru@Ir/C catalysts, the position of the Ru^0^ peak in the case of the Ru@Ir/TiO_*x*_N_*y*_-C sample shows a significant shift of 0.2 eV toward lower BE values. Similarly, the Ir^0^ peak in Ru@Ir/TiO_*x*_N_*y*_-C (Figure 3c) shifts by 0.1 eV to lower BE values compared to Ir/C and Ru@Ir/C, while Ir^4+^ has the same BE value of 61.9 eV as in these two samples. Similar subtle shifts are widely reported in the literature and connected with electronic interactions, either within complex alloy materials^52^ or between metallic active sites and supports.^53,54^

The XPS results can be summarized as follows. In the Ru@Ir/C sample, the peaks for both metallic Ru and Ir are at the same binding energies as in the Ru/C and Ir/C benchmarks. This does not exclude the possibility of interaction between Ru and Ir, but shows that it is not strong enough to be detected by XPS. On the other hand, both the Ru^0^ and Ir^0^ peaks in the spectra of the Ru@Ir/TiO_*x*_N_*y*_-C sample are shifted compared to Ru/C and Ir/C (and Ru@Ir/C), indicating the influence of the TiO_*x*_N_*y*_ support and confirming the rearrangement of electron density on both Ru and Ir sites. Such an effect could adjust the binding energy of the H intermediate species and water activation properties, resulting in enhanced HER activity, which will be investigated further.^19^

### 3.2 Electrocatalytic HER Investigations

#### 3.2.1 Acidic Electrolyte

The activity of Ru@Ir/TiO_*x*_N_*y*_-C and carbon-supported benchmarks was probed first in acidic media, Figure 4. Regarding carbon-supported benchmarks, expected trends can be observed as Pt/C is the most active, followed by Ir/C, while Ru/C shows significantly lower activity, Figure 4a. This is in agreement with the HER Volcano plots, where Pt and Ir are sitting near the Volcano apex due to nearly optimal hydrogen adsorption-free energies, whereas Ru is featured with stronger hydrogen adsorption hindering HER kinetics.^14^ Ru@Ir/C composite is significantly more active than Ru/C, but less active than Ir/C (and Pt/C), indicating the adjustment of hydrogen adsorption-free energy between bare Ru and Ir. Further combining of Ru@Ir active sites with TiO_*x*_N_*y*_ support led to a significant boost in the HER activity, which surpassed Ir/C and nearly matched the activity of the Pt/C. This means that MSI boosted the performance of Ru@Ir sites, indicative of further adjustment of hydrogen adsorption-free energy. The overpotential required to achieve a current density of 10 mA cm^–2^ (η_10_) is a standard descriptor used to compare the HER activity of different catalysts. Comparison of η_10_ values for Ru/C, Ru@Ir/C, Ir/C, Ru/TiO_*x*_N_*y*_-C, and Pt/C in Figure 4b shows values of 97, 26, 18, 15, and 13 mV, respectively. The reported η_10_ values are presented as average values from multiple independent measurements, with error bars representing one standard deviation, confirming the reproducibility and reliability of the obtained data and trends. The mass activity (MA) of the electrocatalysts can provide insights into their practical applicability and efficiency of metal utilization. In terms of MAs, Figure 4c, Ru@Ir/TiO_*x*_N_*y*_-C outperforms all carbon-based benchmarks, including Pt/C, indicating the benefits of optimized nanostructure and MSI. MA values corresponding to the overvoltage of 50 mV are compared in Figure S6. HER kinetics was probed using turnover frequency (TOF) assessment and Tafel slope analysis, Figures 4d and S7. In this work, TOF is calculated assuming that the total loading of the catalysts is involved in the reaction using the following equation:

$$\mathrm{TOF}=\frac{i}{2Fn}$$ 1

where *i* stands for measured HER current, factor 2 reflects two electrons exchanged per H_2_ molecule, *F* is the Faraday constant, and *n* stands for the total number of active sites (i.e., moles of active metal sites). Therefore, as-obtained TOFs refer to the lower limit values; however, they can still be used as a relevant point for catalyst comparison.^55^ TOFs are compared in the whole potential region used for HER screening in Figure S7a, while comparison at η = 50 mV is depicted in Figure 4d. It should be noted that while the TOF values of Ru@Ir/TiO_*x*_N_*y*_-C and Pt/C are comparable in acidic media, the MA of Ru@Ir/TiO_*x*_N_*y*_-C is significantly higher. This difference comes from the normalization approach. TOF calculations assume that all metal atoms are catalytically active, which underestimates the true site-specific activity, particularly in well-dispersed systems. This underestimation is more pronounced for core–shell structured catalysts like Ru@Ir/TiO_*x*_N_*y*_-C (and Ru@Ir/C), where the Ru core contributes a substantial number of atoms per gram of metal due to its low molar mass, yet these active sites are most likely not directly involved in the reaction. In contrast, mass activity more accurately reflects the practical utilization of the loaded metal(s), especially in systems with small, highly dispersed particles and MSI effects.

**Figure 4:**
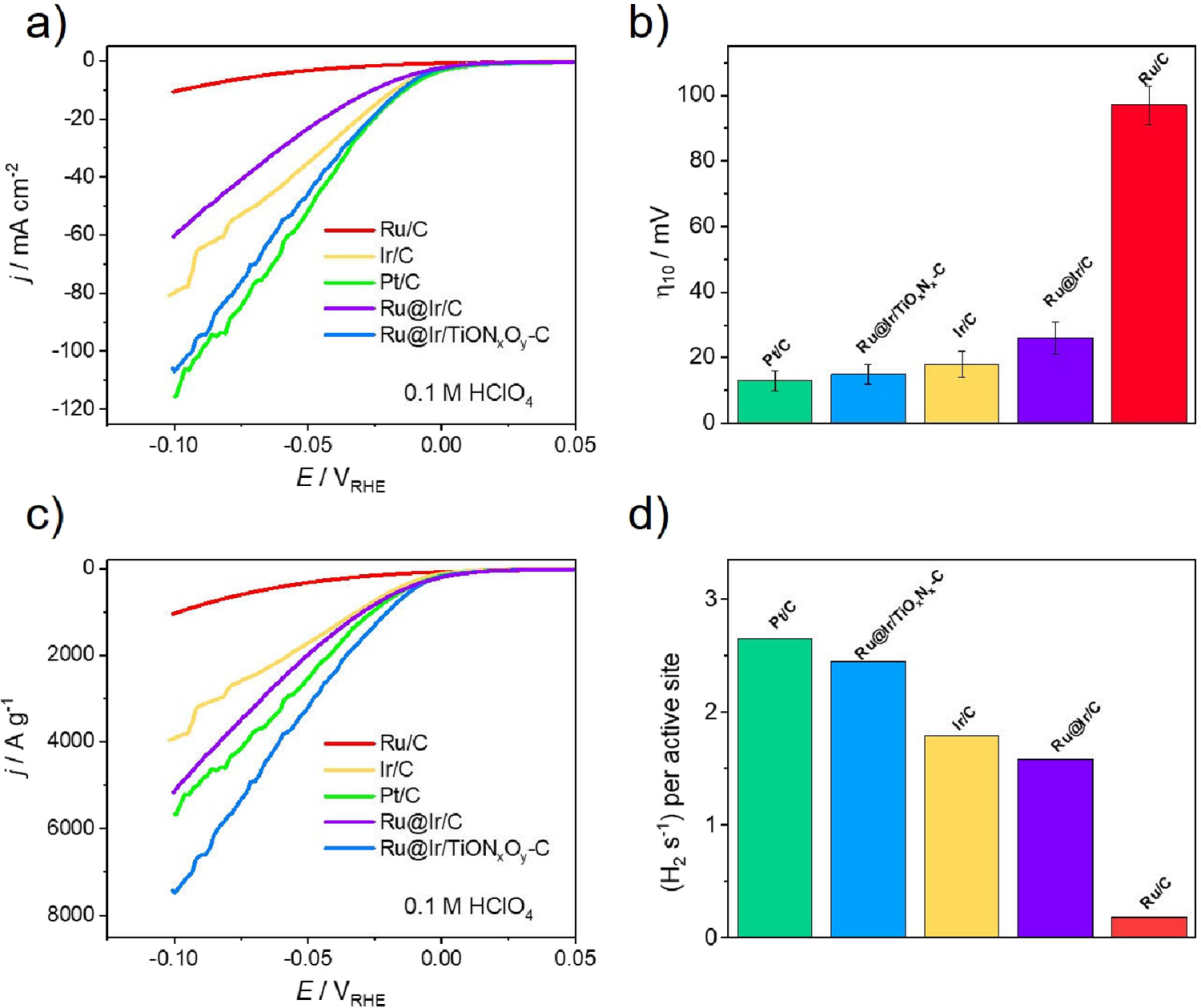
Investigations of HER activity in acid media on Ru@Ir/TiO_*x*_N_*y*_-C, Ru@Ir/C, and carbon-supported benchmarks: (a) HER polarization curves (0.1 M HClO_4_, 10 mV/s); (b) overvoltage at 10 mA cm^–2^ (error bars represent standard deviation from at least three independent measurements); (c) mass activities; and (d) TOFs at an overpotential of 50 mV.

As for the Tafel analysis, Figure S7b, a slope of 30 mV/dec was fitted for Pt/C, Ir/C, and Ru@Ir/TiOxNy-C, while a slightly higher value of 35 mV/dec was obtained for Ru@Ir/C. Such a Tafel slope value is indicative of the Volmer–Tafel pathway, with the Tafel step as the rate-determining step, showing that the adsorption of hydrogen is feasible on these surfaces. In contrast, the Ru/C sample exhibits a high Tafel slope of approximately 80 mV/dec, indicating significantly slower reaction kinetics hampered by strong hydrogen adsorption. Overall, these results show the beneficial effect of combining Ru with Ir to create Ru@Ir nanostructures supported on TiO_*x*_N_*y*_.

The stability of Ru@Ir/TiO_*x*_N_*y*_-C and Ru@Ir/C catalysts was investigated using an extensive potentiodynamic degradation test (Figure 5). It can be seen that Ru@Ir/C is quite unstable in acidic media and suffers a considerable loss of activity after 5000 voltammetric cycles. In contrast, Ru@Ir/TiO_*x*_N_*y*_-C showed stable operation and only slightly decreased activity after 10,000 voltammetric runs in the HER range, showing the positive influence of TiO_*x*_N_*y*_ support on the catalyst’s durability. Additionally, since potentiodynamic and galvanostatic/potentiostatic stability tests could induce different degradation mechanisms, Ru/TiO_*x*_N_*y*_-C was subjected to a constant current density of 10 mA cm^–2^, Figure S8. It is well-known that the formed hydrogen bubbles significantly interfere with galvanostatic/potentiostatic durability tests in the TF-RDE arrangement, often showing activity decay due to the blockage of active sites. We encountered the same problem, as the activity of our catalyst was decreasing rapidly (within minutes), whereas the electrode surface was blocked by visible hydrogen bubbles. To minimize this issue, we diluted five times the catalyst ink to deposit a thinner film that could more easily release hydrogen microbubbles. It can be seen that such Ru/TiO_*x*_N_*y*_-C catalyst film showed quite stable HER operation over a 12-h galvanostatic test. As the main degradation mechanism during HER involves migration, coalescence, and detachment of nanoparticles, it can be suggested that TiO_*x*_N_*y*_ contributes to the durability by stronger anchoring of Ru@Ir active sites compared to carbon, where particles are weakly bonded. This will be investigated in more detail further using DFT calculations.

**Figure 5:**
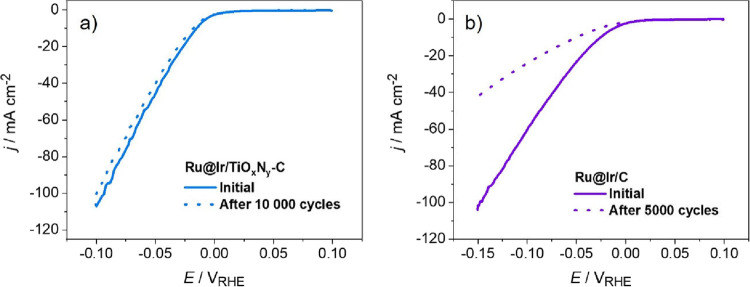
Comparison of the durability of (a) Ru@Ir/TiO_*x*_N_*y*_-C and (b) Ru@Ir/C during potentiodynamic degradation test in acid media (0.1 M HClO_4_, scan rate 100 mV/s, potential range between 0.1 and −0.1 V_RHE_).

#### 3.2.2 Alkaline Electrolyte

The electrocatalytic HER performances of Ru@Ir/TiO_*x*_N_*y*_-C and Ru@Ir/C were further investigated in alkaline media, Figure 6. Both samples show dominant activity in 1 M KOH electrolyte, with the trend following the line Ru@Ir/TiO_*x*_N_*y*_-C > Ru@Ir/C > Pt/C ∼ Ir/C > Ru/C (Figure 6a). A comparison of η_10_ values is given in Figure 6b, showing the impressive value of only 15 mV for Ru@Ir/TiO_*x*_N_*y*_, followed by Ru@Ir/C with 30 mV. Ir/C and Pt/C showed similar η_10_ values (42 and 45 mV, respectively), while Ru/C required slightly more than 100 mV overpotential to reach 10 mA cm^–2^. The error bars represent one standard deviation from a minimum of three replicate measurements, confirming the reproducibility of the data and reliability of observed activity trends. The comparison of HER mass activities in alkaline electrolytes is given in Figures 6c and S9. It can be seen that the Ru@Ir/TiO_*x*_N_*y*_-C sample has the highest MA, followed by the Ru@Ir/C, while carbon-supported benchmarks come with significantly lower mass utilization, Figure 6c. At an overpotential of 100 mV, the MA of Ru@Ir/TiO_*x*_N_*y*_-C is an impressive 8670 A g^–1^, which is more than 6 times higher than that of the Pt/C benchmark (1415 A g^–1^), and significantly higher than Ru/C (998 A g^–1^) and Ir/C (1313 A g^–1^), as depicted in Figure S9. It is worth noting that the MA of Ru@Ir/C is also very high, with the value of 5725 A g^–1^ at an overpotential of 100 mV, which is about 4 times higher than Pt/C. A comparison of intrinsic activities using TOF is given in Figures 6d and S10a. At an overpotential of 100 mV, the Ru@Ir/TiO_*x*_N_*y*_-C sample exhibits a TOF more than 4.5 times higher than the Pt/C benchmark (6.6 vs 1.4 H_2_ s^–1^ per active site), Figure 6d. At the same time, Ru@Ir/C also shows a very high TOF value of 4.4 H_2_ s^–1^ per active site. These trends are valid through the whole potential region used for HER polarization curves, as evident from Figure S10a. The corresponding Tafel analysis is presented in Figure S10b. A Tafel slope of around 40 mV/dec was fitted for Ru@Ir/C, Pt/C, and Ir/C, corresponding to the Volmer-Heyrovsky pathway, whereas Ru@Ir/TiO_*x*_N_*y*_-C shows the Tafel slope of around 20 mV/dec, indicating much faster kinetics and a reaction mechanism change to the Volmer–Tafel route. The highest slope of 60 mV/dec was fitted for Ru/C, indicative of slow reaction kinetics following the Volmer-Heyrovsky pathway with intermediate surface coverages of adsorbed protons.

**Figure 6:**
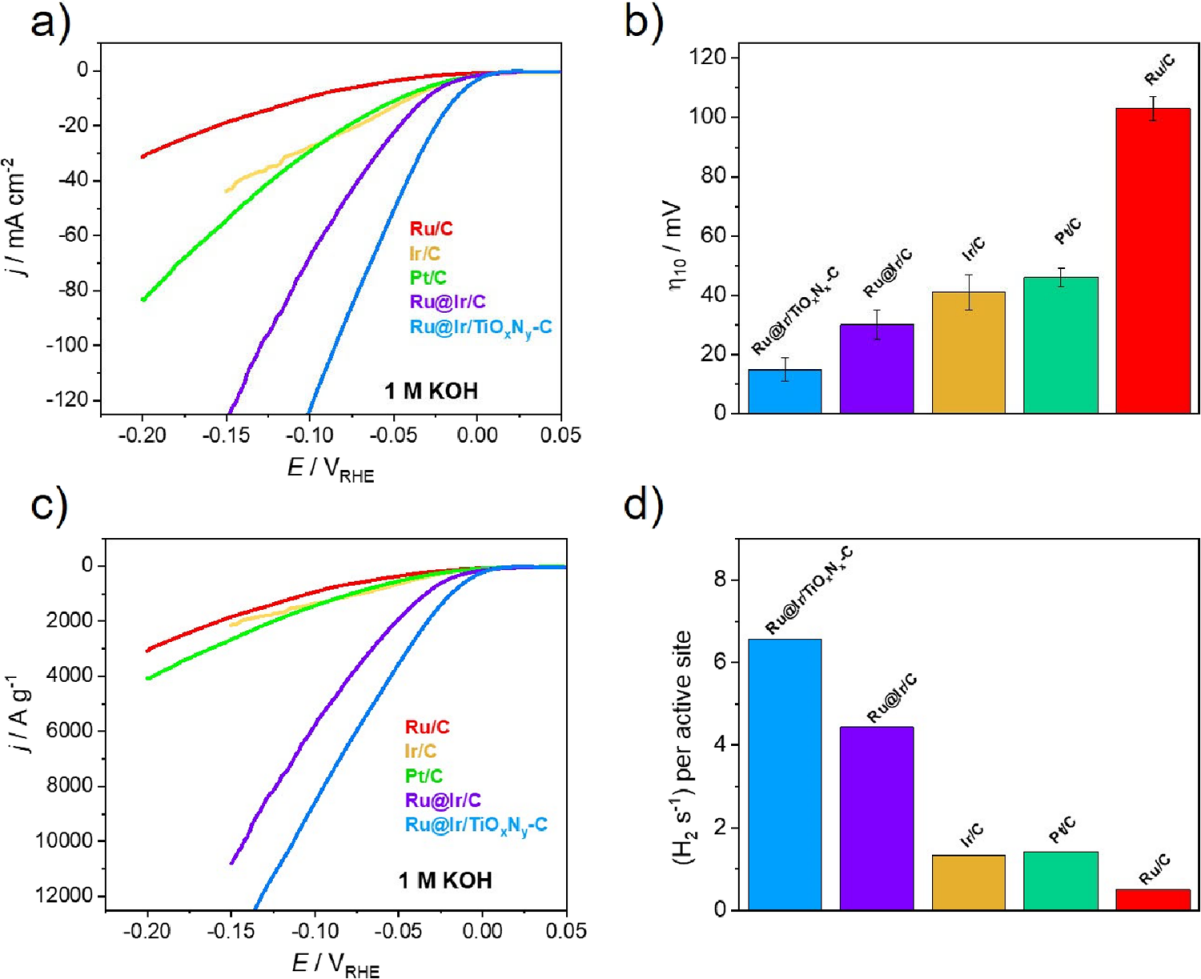
Investigations of HER activity in alkaline media on Ru@Ir/TiO_*x*_N_*y*_-C, Ru@Ir/C, and carbon-supported benchmarks: (a) HER polarization curves (1 M KOH, 10 mV/s); (b) overvoltage at 10 mA cm^–2^ (error bars represent standard deviation from at least three independent measurements); (c) mass activities; and (d) TOFs at an overpotential of 100 mV.

To study the influence of the TiO_*x*_N_*y*_ support on the stability of the catalytic composite, the Ru@Ir/TiO_*x*_N_*y*_-C and Ru@Ir/C catalysts were subjected to different degradation tests, and the obtained results are given in Figure 7. The potentiodynamic test comprised extensive cycles in the HER potential range, Figure 7a,b. The activity of Ru@Ir/TiO_*x*_N_*y*_-C remained completely stable after 10,000 voltammetric scans, while Ru@Ir/C showed a loss of HER activity after the same degradation test. This indicates the positive influence of the TiO_*x*_N_*y*_ support on the durability of the active Ru@Ir sites. Considering previous work with Pt/TiON-C^29^ and Ru/TiON-C, this stability improvement can be attributed to the stronger anchoring of the Ru–Ir nanoparticles on the TiO_*x*_N_*y*_, which suppresses particle migration over support and prevents their detachment and agglomeration as the most common HER degradation mechanisms.^56^

**Figure 7:**
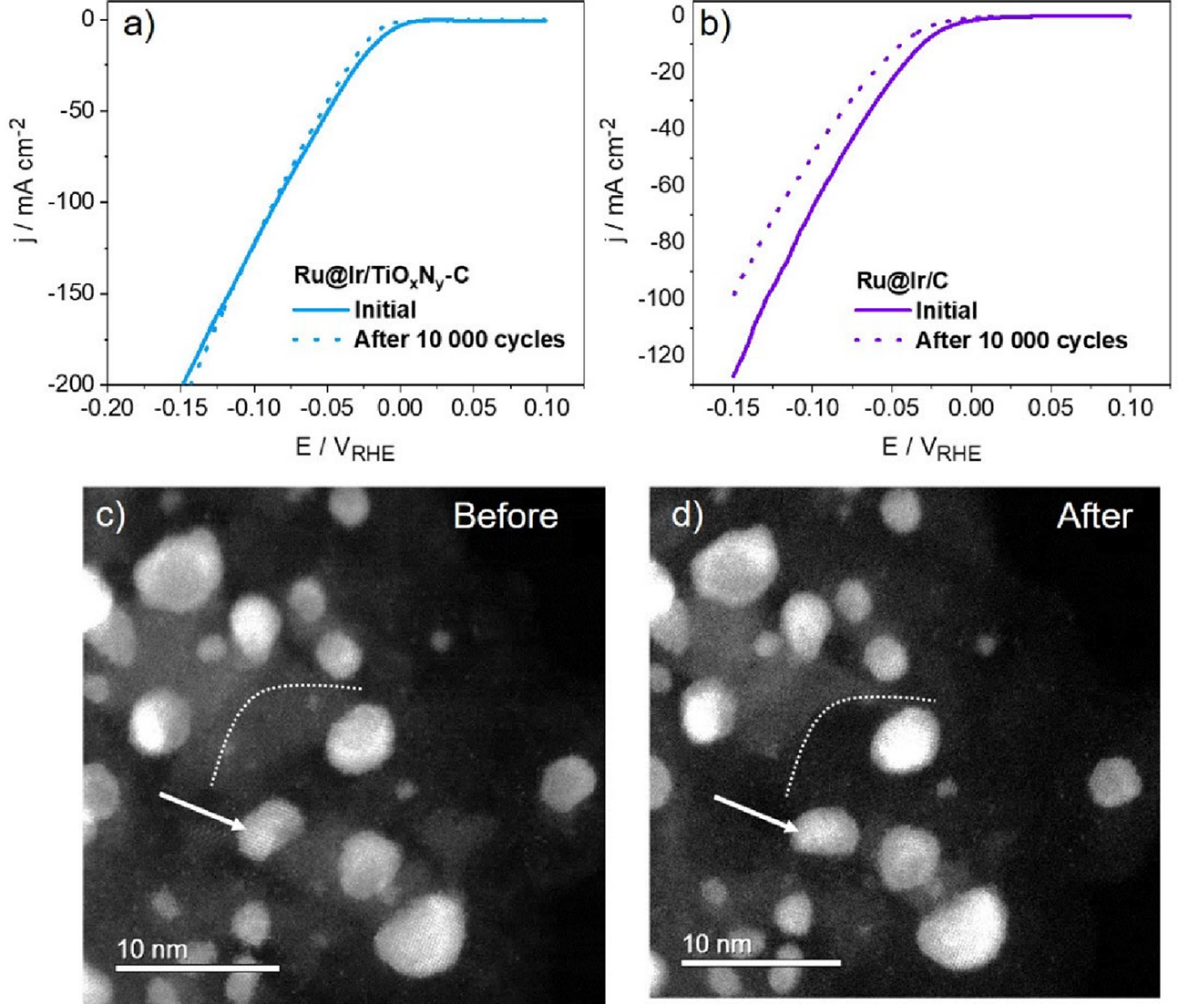
Comparison of HER polarization curves of (a) Ru@Ir/TiO_*x*_N_*y*_-C and (b) Ru@Ir/C before/after degradation test (10,000 sweeps at a scan rate of 100 mV/s in potential region between 0.1 and −0.1 V_RHE_, 1 M KOH); (c,d) IL- STEM ADF imaging of Ru@Ir/TiO_*x*_N_*y*_-C sample before and after potentiostatic durability test (4 h at −0.05 V_RHE_ in 1 M KOH).

To verify the durability of Ru@Ir/TiO_*x*_N_*y*_ -C at the nanoscale, a STEM study was performed at identical locations using an MFE setup, as shown in Figure 7c,d. The IL-STEM ADF analysis confirms the durability of the Ru@Ir/TiO_*x*_N_*y*_-C catalyst in the potentiostatic degradation test (chronoamperometry over 4 h at an overvoltage of 50 mV). The most visible change in the catalyst structure is the physical detachment of a part of the support material (marked by the dashed white line). This could be a nonintrinsic event caused by some external factors as it does not repeat across the catalyst surface. More importantly, the Ru@Ir nanoparticles remained stable as active sites during the degradation test and only minor particle movement was observed (marked by a white arrow).

Finally, it would be interesting to compare the HER performance of Ru@Ir/TiO_*x*_N_*y*_-C with Ru/TiO_*x*_N_*y*_-C sample, which was used as a starting point for engineering a core–shell nanostructure with Ir, Figure S11. It can be seen that in both acid and alkaline electrolytes, significant activity improvement is observed upon addition of Ir, pointing to the synergism between MSI and core–shell nanostructured active sites. The possible origin of the observed catalytic effect will be studied further using DFT calculations.

### 3.3 DFT Calculations

Additional insights into electrocatalytic activity and metal–support interactions were obtained through ab initio modeling based on DFT. Metal electrodes were modeled as 4 × 4 × 1 supercells of 4-atom-layer-thick surface slabs. Extended hcp Ru, fcc Ir, and fcc Pt slabs were constructed parallel to Miller indices yielding the lowest surface energy,^57^ i.e., Ru(0001), Ir(111), and Pt(111). The Ru@Ir bimetallic slab was approximated by replacing the top layer of the Ru(0001) slab with Ir, based on the STEM and EDS analyses, which revealed a nanostructure consisting of an Ir shell and a Ru core.

The HER catalytic activity in acidic conditions was investigated through the change in the Gibbs free energy for hydrogen adsorption Δ*G*_H_ as a descriptor:^58,59^

$$\Delta G_{H}=\frac{1}{n}\left(G_{M-H}-G_{M}-\frac{n}{2}G_{H_{2}}\right)$$ 2

where *G*_M–H_ is the Gibbs free energy of the slab-hydrogen complex, *G*_M_ is the Gibs free energy of the slab, *G*_H_2__ is the Gibbs free energy of a hydrogen molecule, at 300 K and 1 atm, and *n* is the number of adsorbed H. We assume that the entropic and enthalpic contributions of catalyst atoms in the slab to the Gibbs free energy remain negligible (*G*_M_ = *E*_M_). For *G*_M–H_, the vibrational analysis is performed only for the adsorbate atoms.

Furthermore, we employ the computational hydrogen electrode (CHE), where the chemical potential of H^+^/e^–^ in equilibrium is the same as that of 12 H_2_(g) under standard conditions. We sampled 1/16, 1/8, 1/4, 1/2, 3/4, and a full monolayer (ML) (Table S1). Additionally, the theoretical overpotential for HER in acidic conditions (*U*) was estimated^60^ (see SI). The model structure of the Ru@Ir–H complex is illustrated in Figure 8a, while the theoretical HER volcano plot is shown in Figure 8c. Theoretical overpotentials increase as Ru@Ir < Ir < Pt < Ru, suggesting an increased HER activity of Ru@Ir compared to Pt, which can be attributed to the compressive strain experienced by the Ir adlayer.^61^ Conversely, Ru exhibits significantly larger theoretical overpotential than Pt, in accordance with its poor experimental performance in acidic electrolyte (Figure 4). The model does not account for the effects of the support (C or TiO_*x*_N_*y*_-C) on account of sufficiently thick active metal surfaces, solvation,^62^ and electrode potential^63,64^ yet it underscores the importance of Ru–Ir interaction in achieving the experimental electrocatalytic activity of Ru@Ir/TiO_*x*_N_*y*_-C.

**Figure 8:**
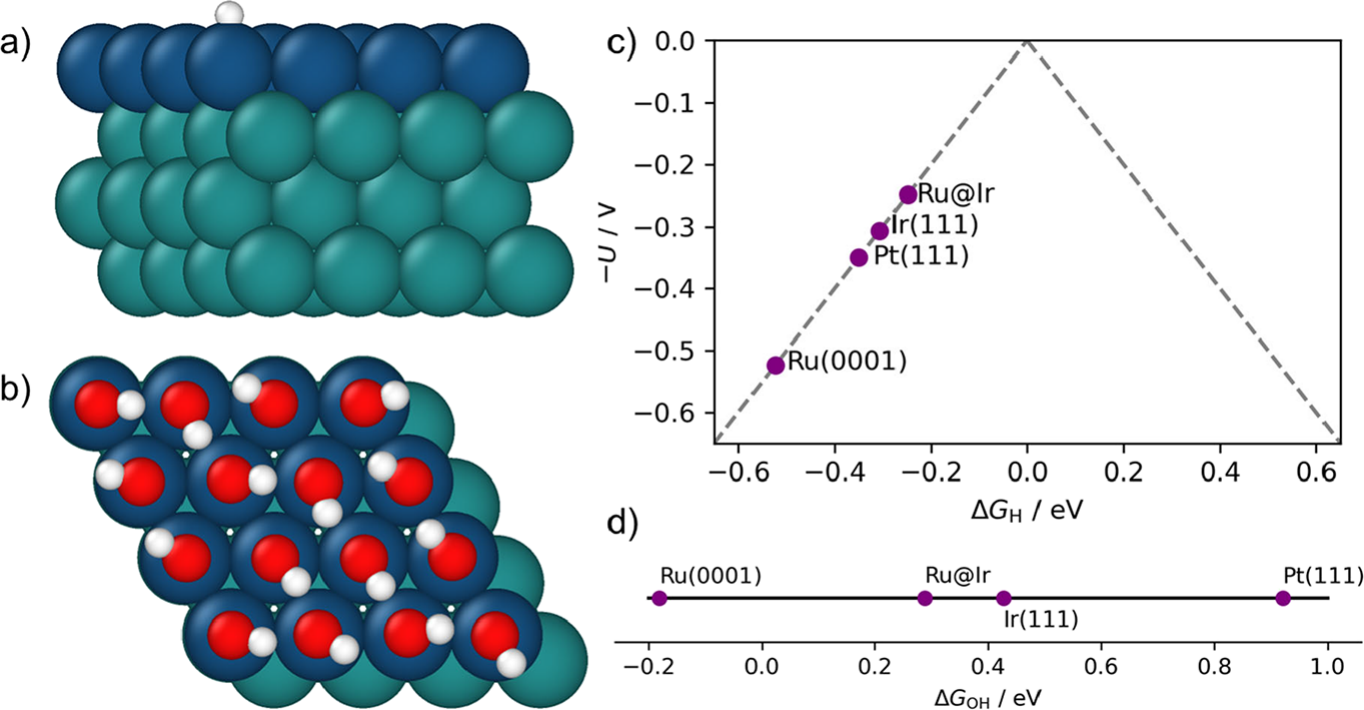
DFT model of H and OH adsorption onto Ru@Ir, Ir, Pt, Ru. Model of Ru@Ir slab with adsorbed (a) O and (b) OH. Ir, Ru, O, and H atoms are shown in blue, green, red, and white, respectively. (c) Volcano plot of theoretical HER overpotential in an acidic medium as a function of the Gibbs free energy of H adsorption onto metal substrates and (d) Gibbs free energy of OH adsorption onto metal substrates.

On the other hand, the HER activity in alkaline media is governed by the adsorption of both H* and OH*.^65^ The change in the Gibbs free energy for OH adsorption was evaluated:

$$\Delta G_{\mathrm{OH}}=\frac{1}{m}\left(G_{M-\mathrm{OH}}+\frac{m}{2}G_{H_{2}}-E_{M}-mG_{H_{2}O}\right)$$ 3

where *G*_M–OH_, *G*_H_2__, and *G*_H_2_O_ are the Gibbs free energy of the slab-hydroxyl complex, a hydrogen molecule, and a water molecule, respectively, all at 300 K and 0.035 atm.^66^ Here, *m* is the number of adsorbed OH in the supercell. Various values of θ were sampled, with the corresponding structures shown in Figure S12. We identified the following surface coverages as the most thermodynamically favorable: 1/8 ML for Pt, 1/2 ML for Ir, 1/16 ML for Ru, and 1/1 for Ir@Ru (Table S2). The model structure of the Ru@Ir–OH complex is illustrated in Figure 8b, and the Δ*G*_OH_ values are given in Figure 8d. In alkaline conditions, the highest rates of hydrogen evolution are observed for catalysts that strongly bind OH and bind H at a strength near 0 eV (relative to H_2_(g)).^65^ The Gibbs free energy of OH adsorption increases (from most to least strongly bound) as Ru < Ru@Ir < Ir < Pt. While Ru binds OH strongly, its strong interaction with H (Figure 8c) is not suitable for high HER rates. Ru@Ir, on the other hand, binds OH strongly and H weakly enough, explaining its high HER experimental electrocatalytic performance in the alkaline medium (Figure 6). A weaker binding of OH to Ru@Ir compared to Ir is surprising: the lattice strain in Ru@Ir would imply a stronger binding, as observed for H. We speculate that this could be attributed to Ru–Ir interaction modulating the electronic structure of the Ir layer. Indeed, Bader charge analysis^67^ reveals a significant charge transfer from Ru to Ir, with Ir atoms gaining −0.16*e*_0_ surplus charge. The partial transfer of electronic density from Ru to Ir is also evident in the differential charge density distribution (Figure S13). The large difference in the Δ*G*_OH_ between Ru@Ir and Pt, compared to a relatively smaller difference in Δ*G*_H_, could explain larger differences in the experimental performance of catalysts in the alkaline medium, compared to acidic, and suggests that the strong OH binding to Ru@Ir is the origin of outstanding electrocatalytic performance of Ru@Ir/TiO_*x*_N_*y*_-C in alkaline conditions.

We inspected the effect of the support, whose interaction with the metal is crucial for metal dispersion and, ultimately, catalyst performance. To gain an insight into the interaction between the metal and support, we studied the adsorption of Ir and Ru atoms onto C and TiO_*x*_N_*y*_. The adsorption energy Δ*E*_ad_ was computed as

$$\Delta E_{\mathrm{ad}}=E_{s-a}-E_{a}-E_{s}$$ 4

where *E*_s–a_, *E*_a_, and *E*_s_ are ground state electronic energies of support-adsorbate complex, adsorptive, and support, respectively. Here, the adsorbate is an Ir or Ru atom, while the carbon surface is approximated as graphene, and TiO_*x*_N_*y*_ surface structure is constructed following our previous work^35^ and provided in Supporting Information. Adsorption was evaluated by sampling a 15 × 15 grid within one unit cell of 4 × 4 × 1 and 2 × 2 × 1 supercell for graphene and TiO_*x*_N_*y*_, respectively, to obtain the most favorable adsorption position and the barriers for surface diffusion, which govern sintering. Figure 9 shows the results of adsorption energy mapping. On the left, unit cell structures of graphene and TiO_*x*_N_*y*_ surface slabs are shown. In the middle and on the right, Ir and Ru adsorption energy maps are shown, respectively. Adsorption of both Ir and Ru onto graphene is substantially weaker than onto titanium oxynitride, similar to the previous observation for Pt.^29^ A stronger interaction between the support and metal catalyst, such as observed with TiO_*x*_N_*y*_, results in a weaker interaction with small species,^68^ which could reduce overpotential for HER. Additionally, stronger support–catalyst interaction maximizes the catalyst surface area,^29^ further improving the catalytic performance. Additionally, Ru atoms adsorb to both graphene and TiO_*x*_N_*y*_ more strongly than Ir atoms. This rationalizes our synthetic approach, i.e., the deposition of strongly adsorbing Ru onto a support, followed by the deposition of Ir, which interacts with the support more weakly. The barrier for surface diffusion is up to 2 orders of magnitude greater on TiO_*x*_N_*y*_ (3.6 and 3.7 eV for Ir and Ru) than on graphene (0.03 and 0.09 eV for Ir and Ru). This impedes the sintering of metallic particles on TiO_*x*_N_*y*_, leading to the formation of smaller nanoclusters, in line with particle size distributions observed with STEM (Figures 1 and S2–s4), which is favorable for the electrocatalytic process.

**Figure 9:**
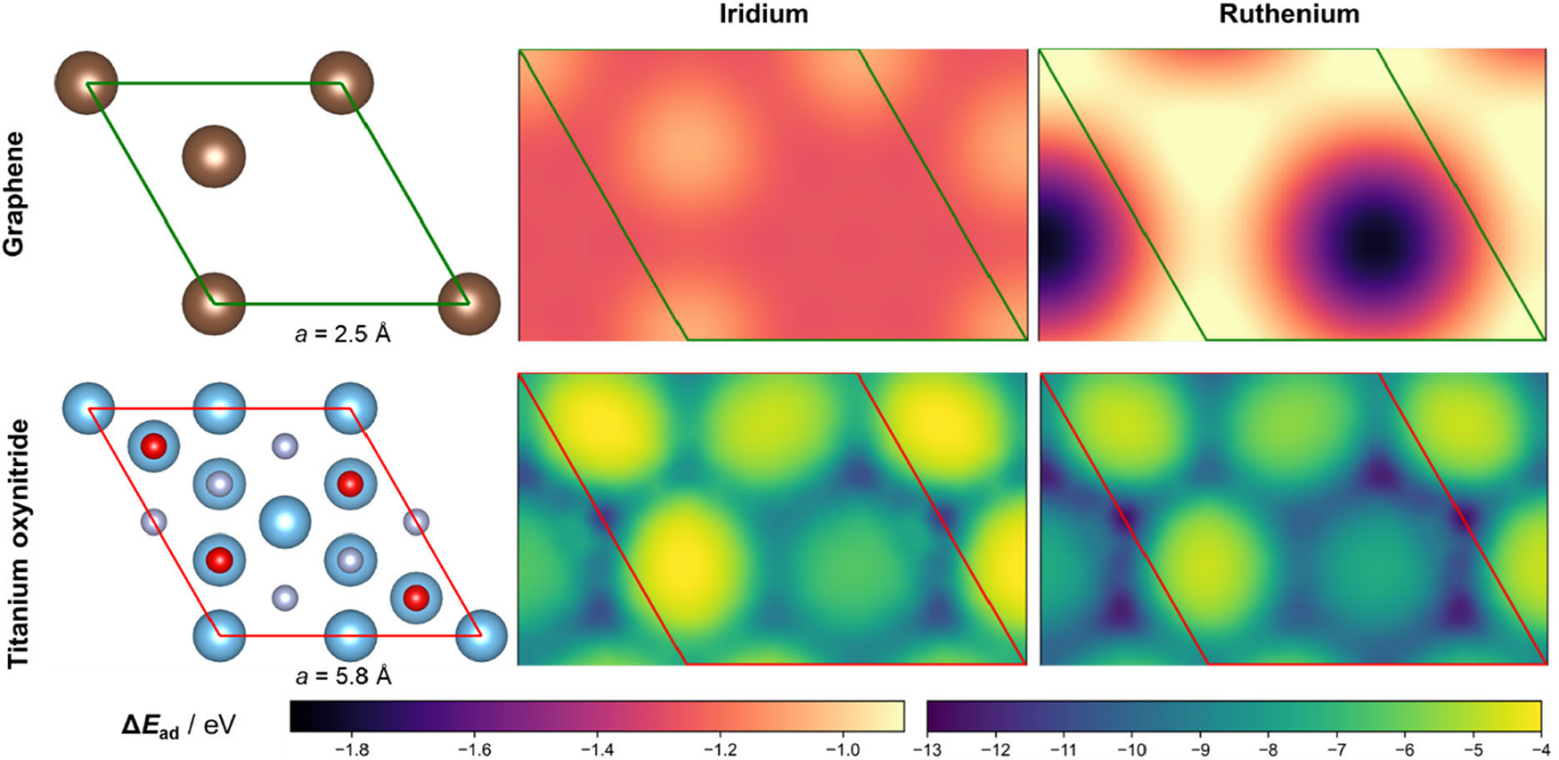
Structures of graphene (top) and titanium oxynitride surface slab (bottom) unit cells (left) and adsorption energies for adsorption of Ir (middle) and Ru (right) atoms. a indicates the unit cell parameter. The magnitude of the adsorption energy is shown with a color map. Dark and light colors correspond to strong and weak adsorption, respectively.

Lastly, we investigated the effect of support on the electronic structure of the electrocatalyst. Specifically, we studied the electronic structure of 5-atom clusters, Ir_5_ and Ru_5_, supported on graphene or TiO_*x*_N_*y*_. Model Ir_5_ and Ru_5_ clusters adsorbed onto both supports are illustrated in Figure 10a. Ir/Ru partial electronic densities of states of adsorbed clusters are shown in Figure 10b. For both clusters, a substantial increase in the density of electronic states of the cluster near the Fermi level is observed when the cluster is adsorbed to TiO_*x*_N_*y*_, compared to C. An increased density of states in the vicinity of the Fermi level facilitates electron transport and is often related to the high activity of the electrocatalysts.^69^

**Figure 10:**
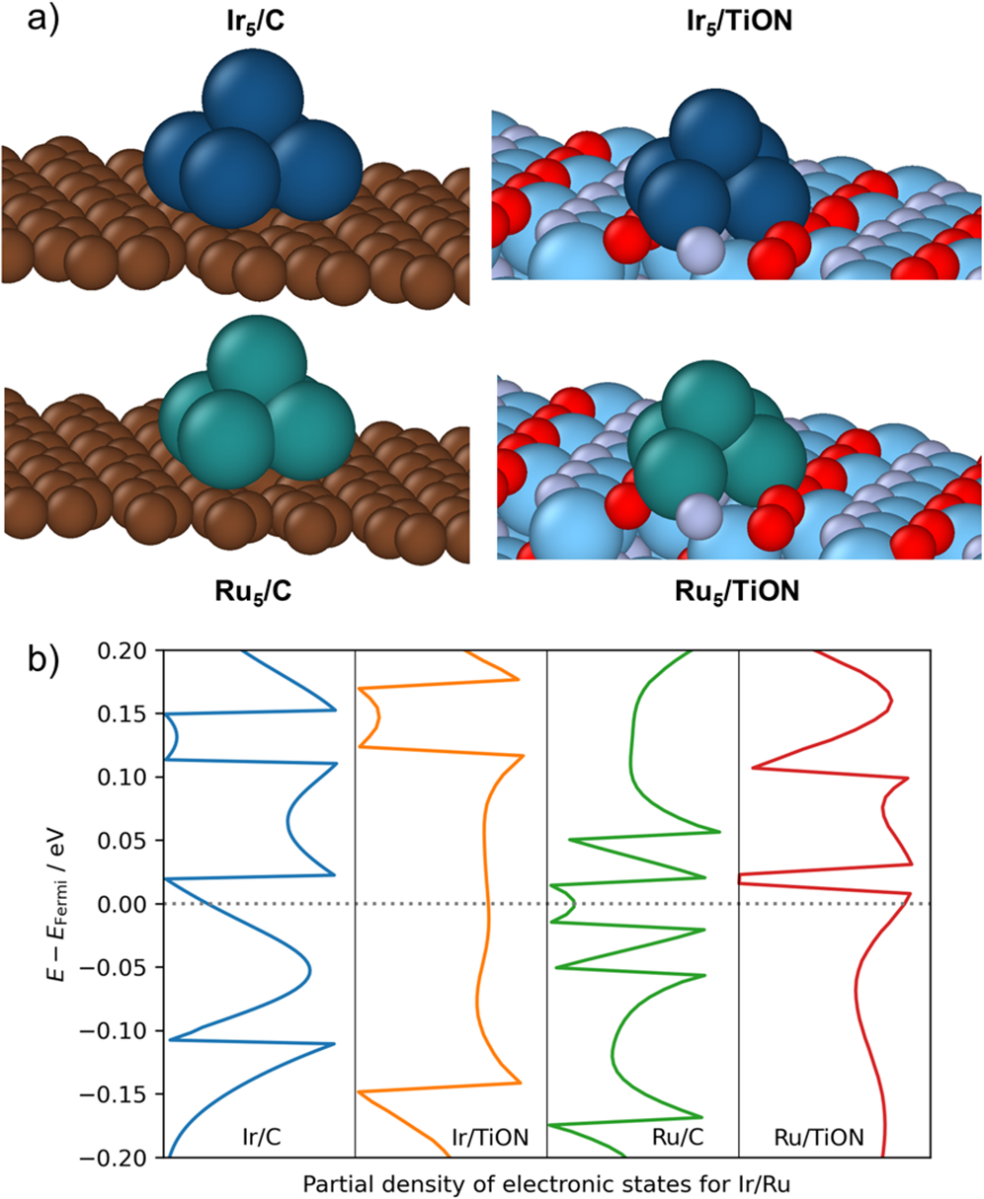
Adsorption of Ir_5_ and Ru_5_ clusters onto carbon and titanium oxynitride support: (a) model of iridium and ruthenium clusters adsorbed onto both supports and (b) Ir/Ru partial densities of electronic states for five-atom clusters adsorbed onto support.

The experimental and theoretical findings supporting the pH-universal performance of the Ru@Ir/TiO_*x*_N_*y*_-C catalyst for the HER are summarized as follows. In acidic media, the HER on highly active catalysts such as Pt/C and Ru@Ir/TiO_*x*_N_*y*_-C proceeds primarily via the Volmer–Tafel mechanism:

$$\mathrm{Volmer}\;\mathrm{step}:\;S+H^{+}+e^{-}\to S-H_{\mathrm{ads}}$$ 5

$$\mathrm{Tafel}\;\mathrm{step}:\;2S-H_{\mathrm{ads}}\to H_{2}+2S$$ 6

where *S* stands for active site. The excellent activity of Pt arises from its nearly optimal Gibbs free energy for hydrogen adsorption (Δ*G*_H_). Our DFT calculations show that Ru@Ir active sites possess similarly favorable hydrogen adsorption properties, indicating that the core–shell architecture effectively tunes Δ*G*_H_, mainly due to the significant electron transfer from Ru to Ir. This can be further modulated by the MSI with TiO_*x*_N_*y*_, enabling HER activity in acidic media that is on par with Pt/C. In alkaline electrolytes, HER is more complex due to the additional water dissociation step, which precedes hydrogen adsorption:

$$\mathrm{Volmer}\;\mathrm{step}:\;S+H_{2}O+e^{-}\to S-H_{\mathrm{ads}}+{\mathrm{OH}}^{-}$$ 7

In this case, the Gibbs free energy of OH adsorption (Δ*G*_OH_) appears as another key activity descriptor, since the presence of OH species can facilitate water dissociation.^65^ Our DFT calculations indicate that Ru@Ir sites bind OH more strongly than Pt, which facilitates the Volmer step as the usual rate-limiting step under alkaline conditions. At the same time, hydrogen binding remains near optimal, resulting in exceptional alkaline HER activity of the Ru@Ir/TiO_*x*_N_*y*_-C catalyst. This dual optimization of H and OH adsorption energies, as well as catalyst stability, can be further boosted by MSI provided by TiO_*x*_N_*y*_ support, resulting in the excellent pH-universal HER performance of Ru@Ir/TiO_*x*_N_*y*_-C composite.

## 4 Conclusions

In conclusion, we have demonstrated a highly efficient and durable hydrogen evolution electrocatalyst based on Ru@Ir core–shell nanoparticles supported on a conductive titanium oxynitride–graphene hybrid (Ru@Ir/TiO_*x*_N_*y*_-C). The combination of core–shell architecture and strong metal–support interaction significantly enhances catalytic activity and long-term stability across the entire pH range. In alkaline media, the catalyst surpasses commercial Pt/C in both activity and mass utilization, while in acidic conditions it matches Pt/C and outperforms monometallic counterparts. XPS analysis and DFT calculations reveal the cumulative effects of Ru–Ir interface and MSI with TiO_*x*_N_y,_ optimizing the adsorption energetics of H and OH intermediates, underpinning the superior HER performance. Moreover, the strong interaction between the TiO_*x*_N_*y*_ support and metal nanoparticles contributes to structural integrity during prolonged operation, minimizing agglomeration and detachment of nanoparticles. This work highlights a generalizable design strategy that integrates advanced conductive supports with tailored nanostructures to enable high-performance, low-PGM electrocatalysts. The approach presented here can be readily extended to other metal combinations and energy conversion reactions, offering a promising pathway toward sustainable and cost-effective technologies.

## Supplementary Material



## Data Availability

The data supporting this article have been included as part of the SI.

## Notes

Characterization of the Ru@Ir/C sample; additional characterization of the Ru@Ir/TiO_*x*_N_*y*_-C sample; XPS survey spectra for Ru/C, Ir/C, Ru@Ir/C, and Ru@Ir/TiO_*x*_N_*y*_-C; additional HER performance analysis in acid and alkaline media and durability tests; and additional DFT calculation data (H adsorption to surface slabs, OH adsorption to surface slabs, estimation of theoretical overpotential for HER, differential charge density for Ru@Ir, and structure of TiO_*x*_N_*y*_ surface slab) (PDF)
