# Supplementary material for: Engineering Ru@Ir Core–Shell Nanoparticles on Titanium Oxynitride–Graphene Support for a Highly Active and Durable pH-Universal Hydrogen Evolution Reaction
Source: ACS Catal. 2025 Jul 21;15(15):13444–57. doi: 10.1021/acscatal.5c02831 (PMC12322918; doi:10.1021/acscatal.5c02831)
Supplement: Supplementary file 1 [file cs5c02831_si_001.pdf]

Supporting information

Engineering Ru@Ir Core-Shell Nanoparticles on Titanium Oxynitride–Graphene Support for Highly Active and Durable pH-Universal Hydrogen Evolution Reaction

A. Popović[a,b], I. Marić[a,c], M. Bele[a], E. Rems[a,d], M. Huš[e,f,g], L. Pavko[a], F. Ruiz-Zepeda[a,h], L. Bijelić[a], B. Grgur[b], N. Hodnik[a,h,i,j], M. Smiljanić[a]

[a] *Department of Materials Chemistry, National Institute of Chemistry, Hajdrihova 19, 1000 Ljubljana, Slovenia*

[b] *Faculty of Technology and Metallurgy, University of Belgrade, Karnegijeva 4, 11000 Belgrade, Serbia*

[c] *Radiation Chemistry and Dosimetry Laboratory, Division of Materials Chemistry, Ruđer Bošković Institute, Bijenička 54, 10000 Zagreb, Croatia*

[d] *Faculty of Chemistry and Chemical Technology, University of Ljubljana, Večna pot 113, 1000 Ljubljana, Slovenia*

[e] *Department of Catalysis and Chemical Reaction Engineering, National Institute of Chemistry, Hajdrihova 19, 1000 Ljubljana, Slovenia*

[f] *Association for Technical Culture of Slovenia (ZOTKS), Zaloška 65, 1000 Ljubljana, Slovenia*

[g] *Institute for the Protection of Cultural Heritage of Slovenia (ZVKDS), Poljanska 40, 1000 Ljubljana, Slovenia*

[h] *Department of Physics and Chemistry of Materials, Institute of Metals and Technology, Lepi pot 11, 1000 Ljubljana, Slovenia*

[i] *Jožef Stefan International Postgraduate School, Jamova cesta 39, 1000 Ljubljana, Slovenia*

[j] *University of Nova Gorica, Vipavska 13, 5000 Nova Gorica, Slovenia*

[*]Corresponding authors: milutin.smiljanic@ki.si; nejc.hodnik@ki.si

Characterization of Ru@Ir/C sample

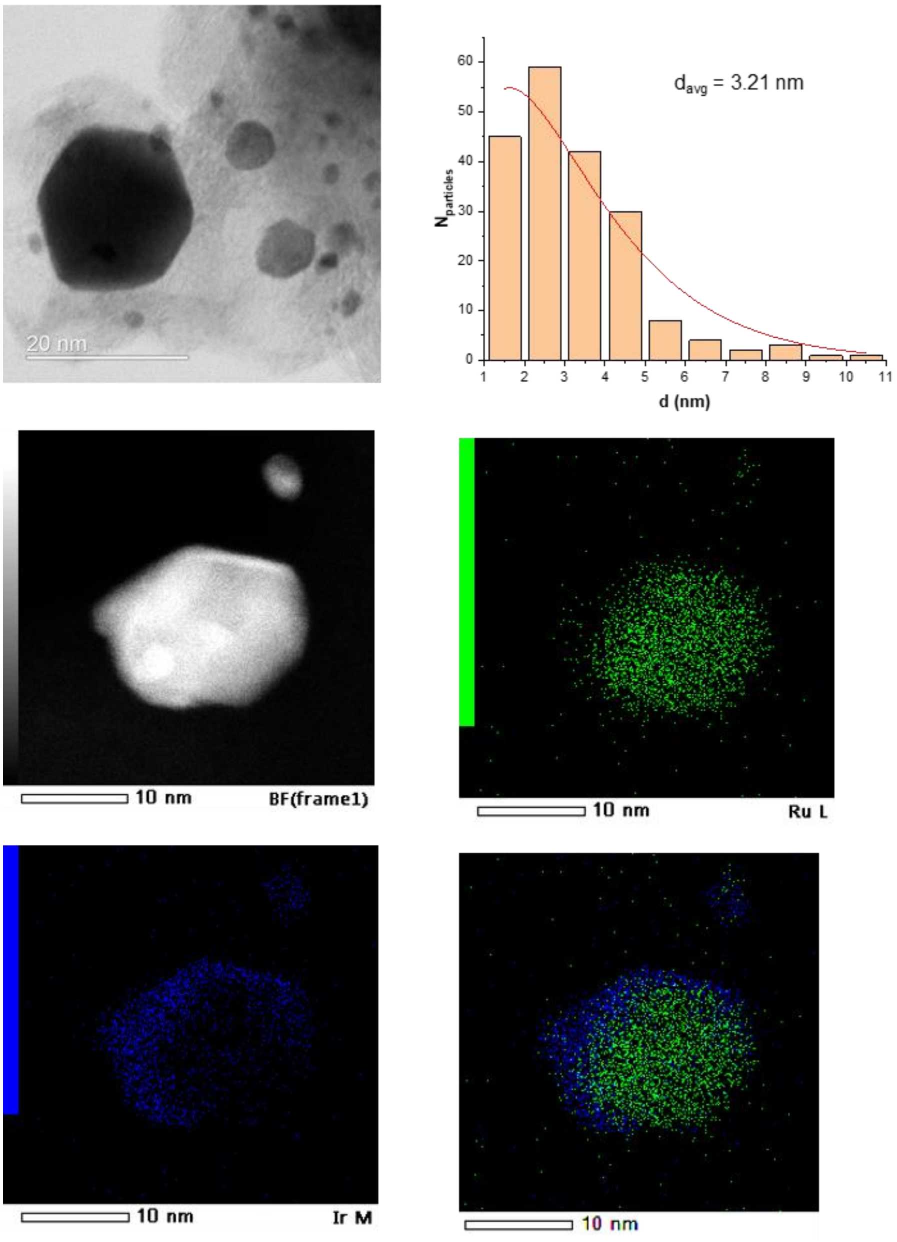

Figure S1. STEM BF and ADF imaging, along with EDS maps and particle size distribution of Ru@Ir/C catalysts.

Additional characterization of the Ru@Ir/Ti$O_x$$N_y$-C sample

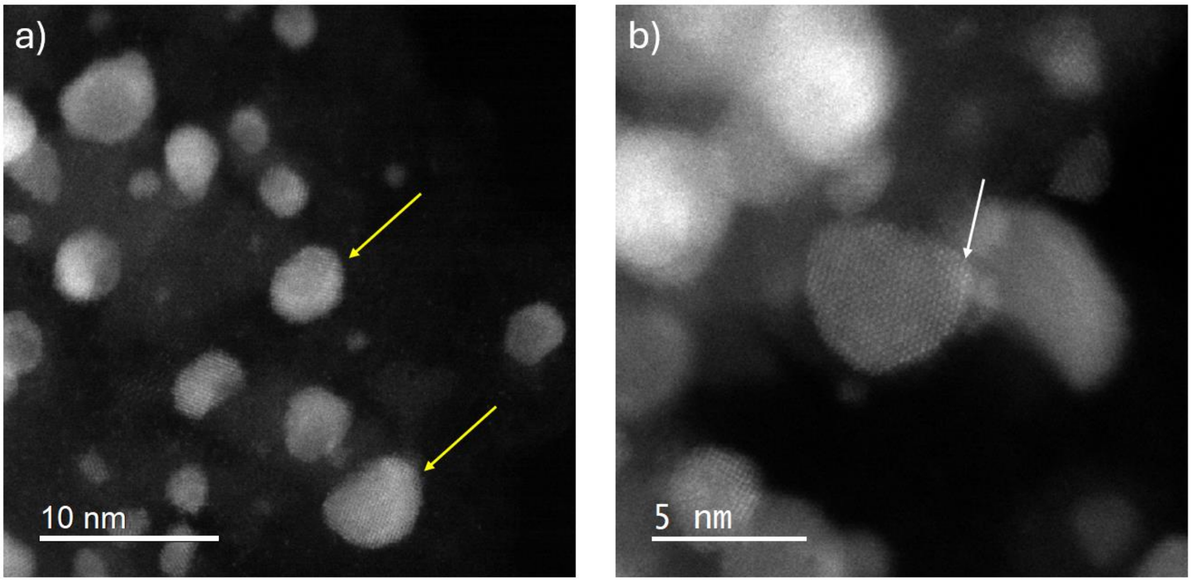

Figure S2. ADF images of Ru@Ir core-shell nanoparticles on Titanium Oxynitride–Graphene support. (a) The two particles marked with yellow arrows are close to zone axis, illustrating a coherent interface between the core (Ru=44) and the shell (Ir=77). (b) A particle close to zone axis with a very thin shell, almost two monolayers thick, as indicated by the arrow.

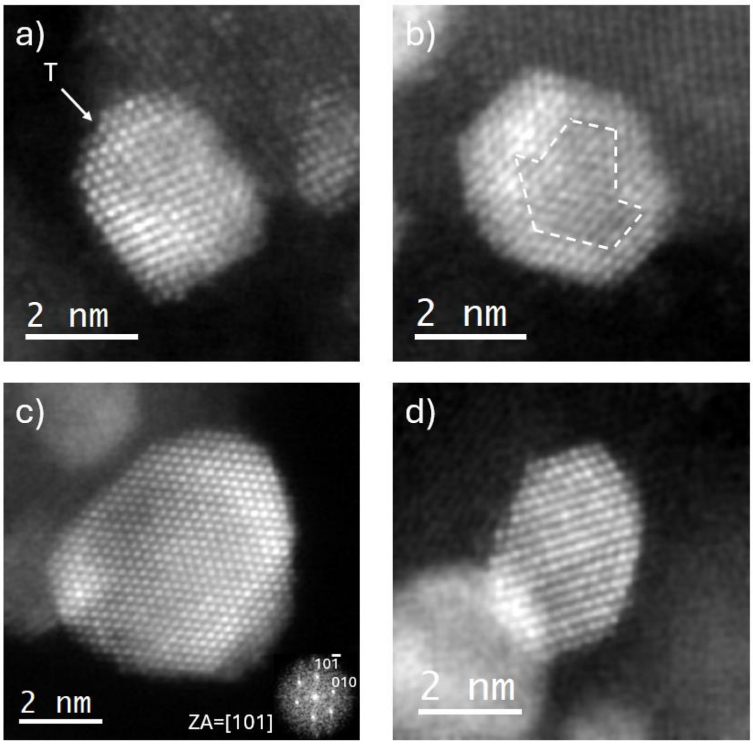

Figure S3. HR ADF imaging of Ru@Ir core-shell nanoparticles on Titanium Oxynitride–Graphene support showing a coherent interface structure between the core and the shell. (a) A core-shell nanoparticle with a twin defect on one side (marked with an arrow). (b) A core-shell nanoparticle where the interface between the core (Ru=44) and the shell (Ir=77) can be seen due to the difference in contrast, as roughly marked with a dashed line. (c) A high coherence at the interface between the core and the shell can be observed in the nanoparticle imaged with Z contrast on the zone axis [101]. (d) A nanoparticle slightly off-axis, where a more uneven shell is observed.

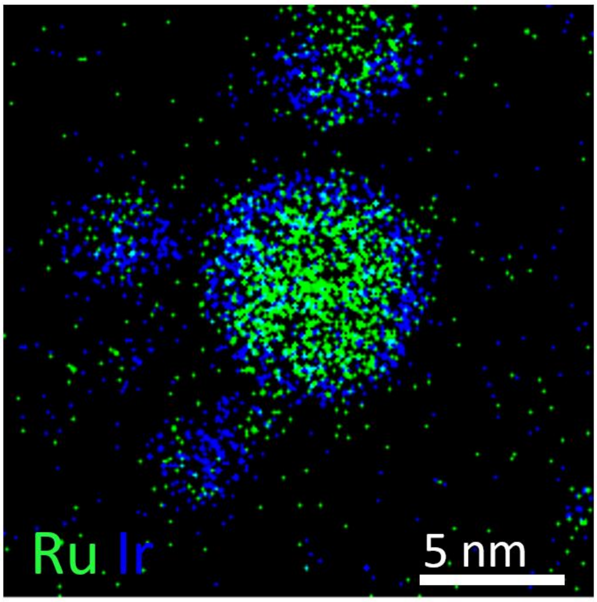

Figure S4. EDS map of Ru@Ir core-shell nanoparticles on Titanium Oxynitride–Graphene support displaying the Ru (green) and Ir (blue) signals.

Survey spectra for Ru/C, Ir/C, Ru@Ir/C, and Ru@Ir/TiO$_x$N$_y$-C

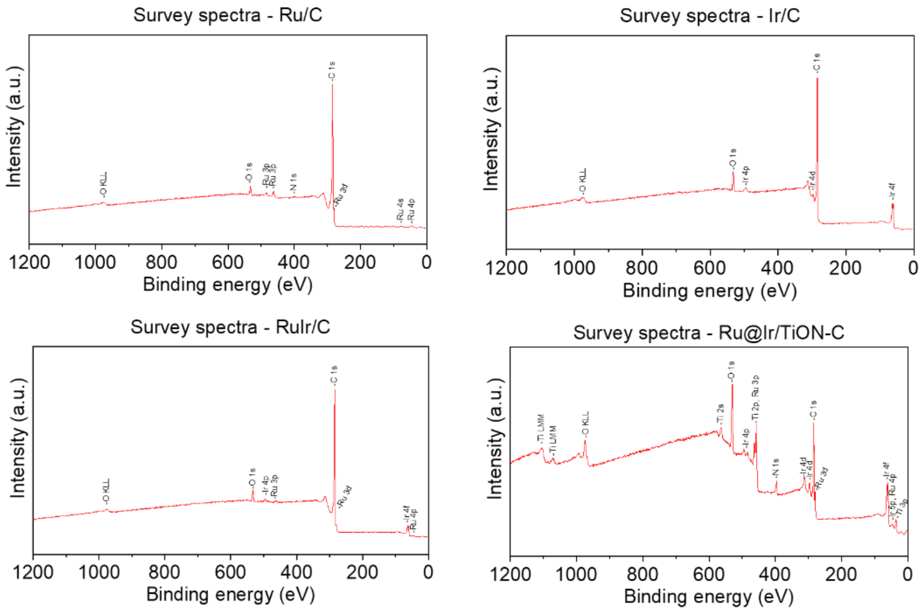

Figure S5. XPS survey spectra for Ru/C, Ir/C, Ru@Ir/C, and Ru@Ir/TiO$_x$N$_y$-C samples.

Mass activity comparison in acid media

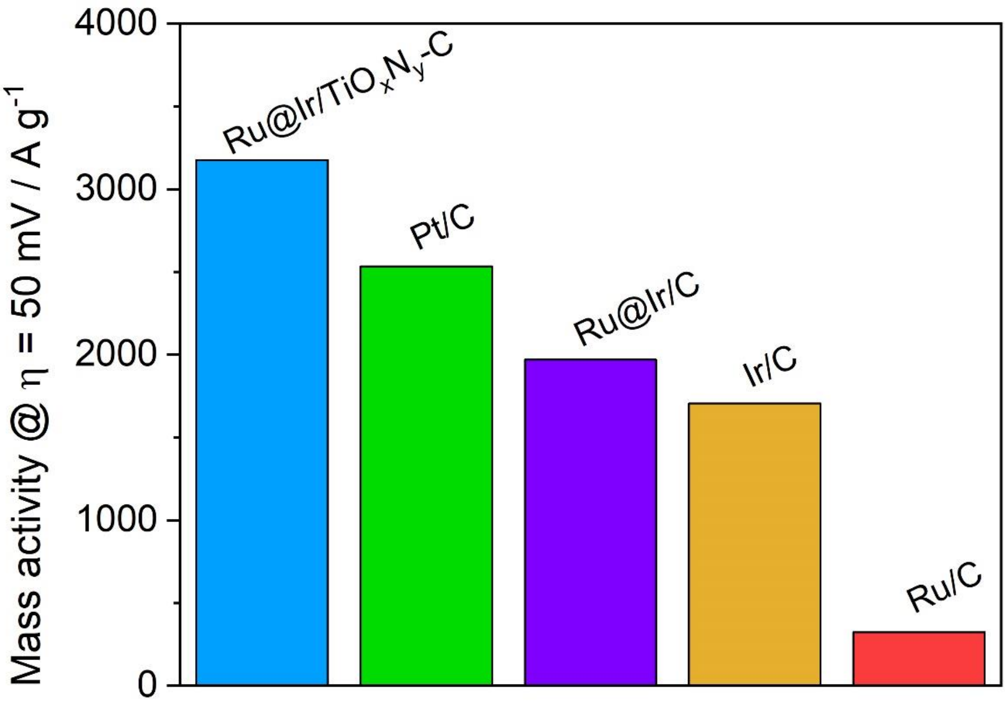

Figure S6. Comparison of mass activities of Ru/C, Ir/C, Pt/C, Ru@Ir/C, and Ru@Ir/TiO_xN_y-C catalysts in acid media at an overpotential of 50 mV (derived from polarization curves presented in Figure 4).

TOF comparison and Tafel slope analysis for HER investigations in acid media

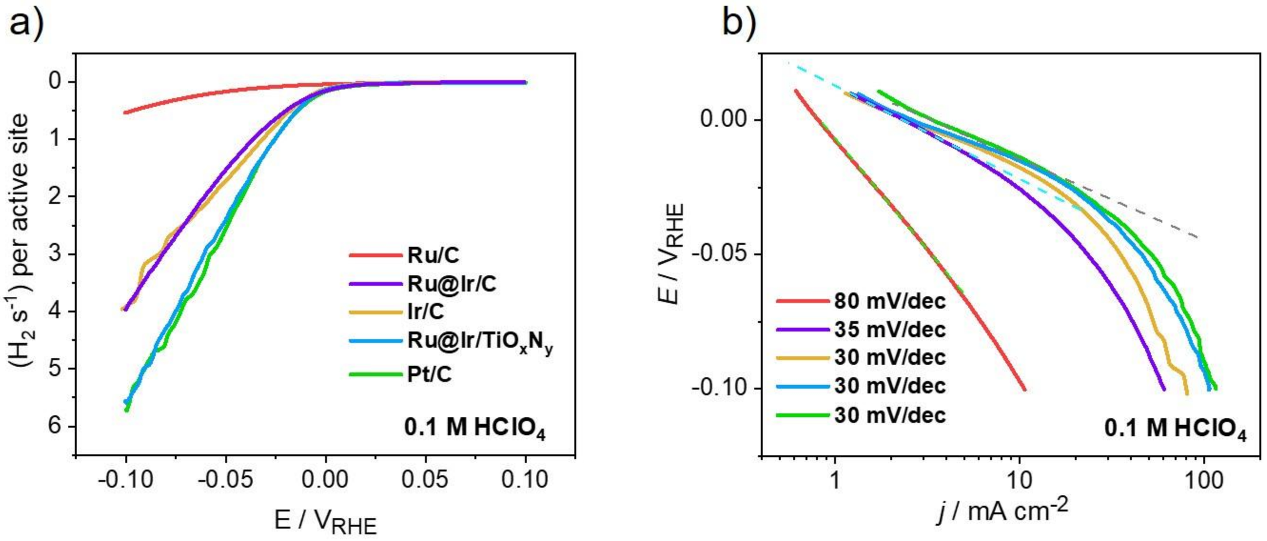

Figure S7. (a) TOF comparison and (b) Tafel slope analysis for Ru/C, Ir/C, Pt/C, Ru@Ir/C and Ru@Ir/TiO_xN_y-C catalysts in acid media obtained from polarization curves from Figure 4 (0.1 M $HClO_4$, 10 mV/s).

Galvanostatic durability test in acid media

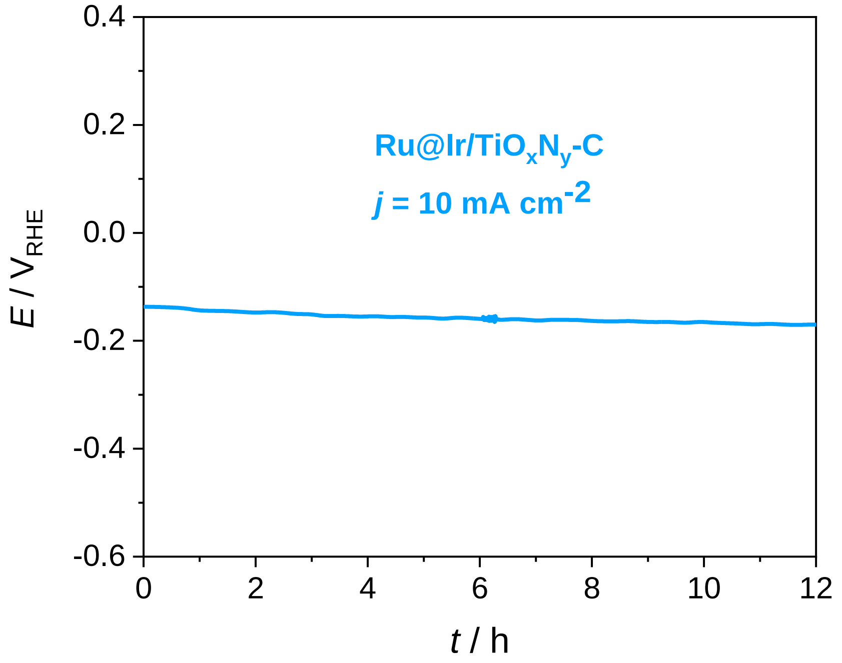

Figure S8. Galvanostatic durability test of Ru/TiO$_x$N$_y$-C in acid electrolyte (0.1 M $HClO_4$, j = 10 mA cm^{-2}). Catalyst loading is five times lower than the loading used to investigate activity (Figure 4) and stability in potentiodynamic conditions (Figure 5).

Mass activity comparison in alkaline media

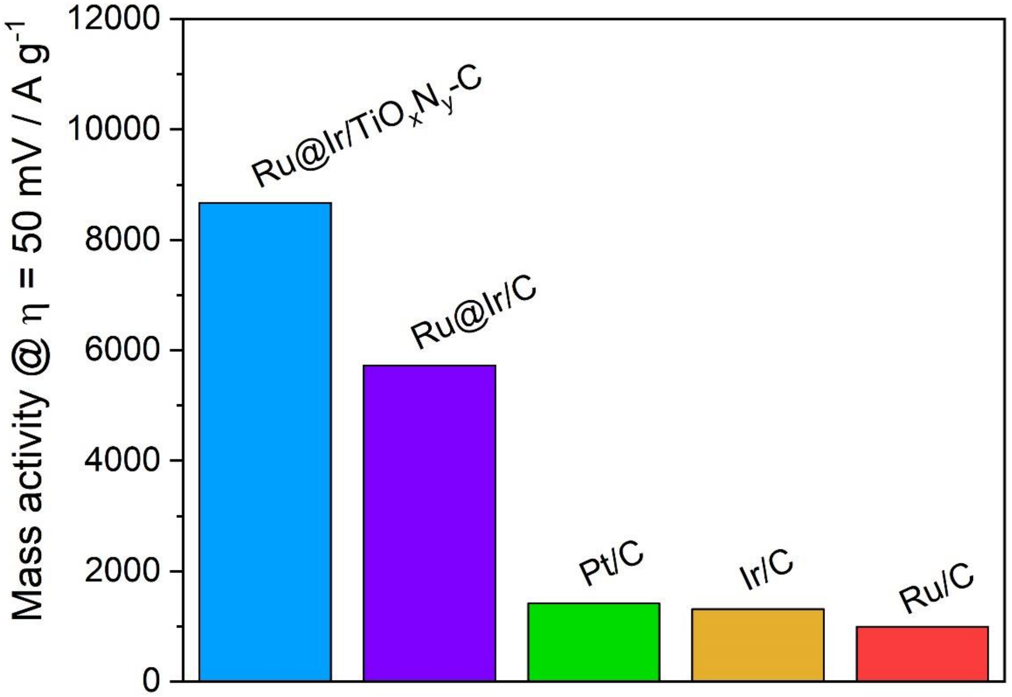

Figure S9. Comparison of mass activities of Ru/C, Ir/C, Pt/C, Ru@Ir/C, and Ru@Ir/TiOxNy-C catalysts in alkaline media at an overpotential of 100 mV (derived from polarization curves presented in Figure 6).

TOF comparison and Tafel slope analysis for HER investigations in alkaline media

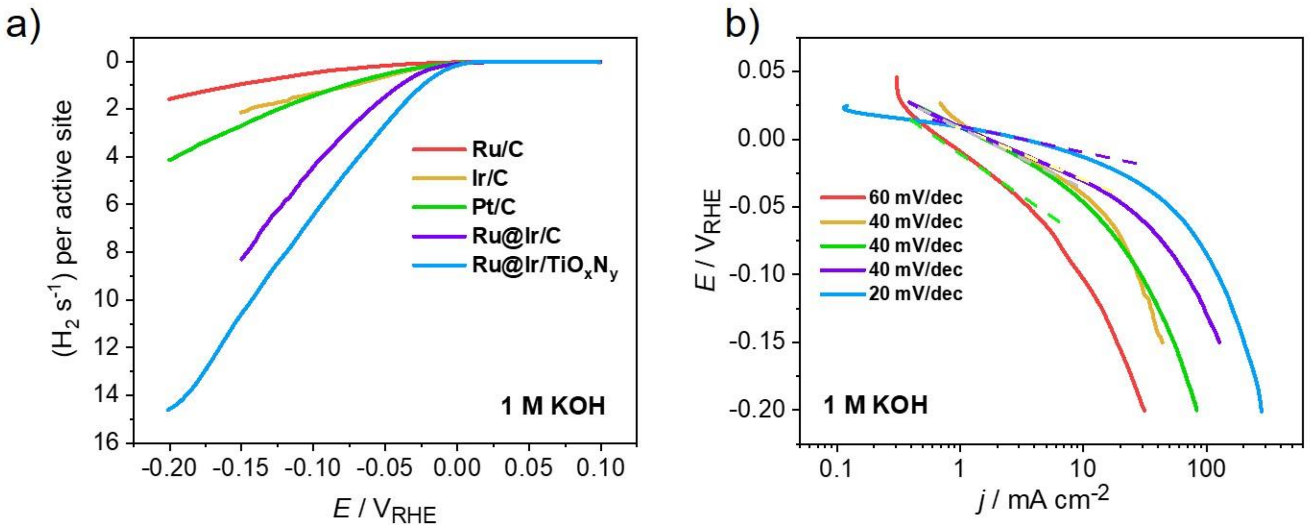

Figure S10. (a) TOF comparison and (b) Tafel slope analysis for Ru/C, Ir/C, Pt/C, Ru@Ir/C and Ru@Ir/TiO_xN_y-C catalysts in acid media obtained from polarization curves from Figure 6 (1 M KOH, 10 mV/s).

Comparison of HER performance of Ru@Ir/TiO_xN_y-C and Ru/TiO_xN_y-C samples

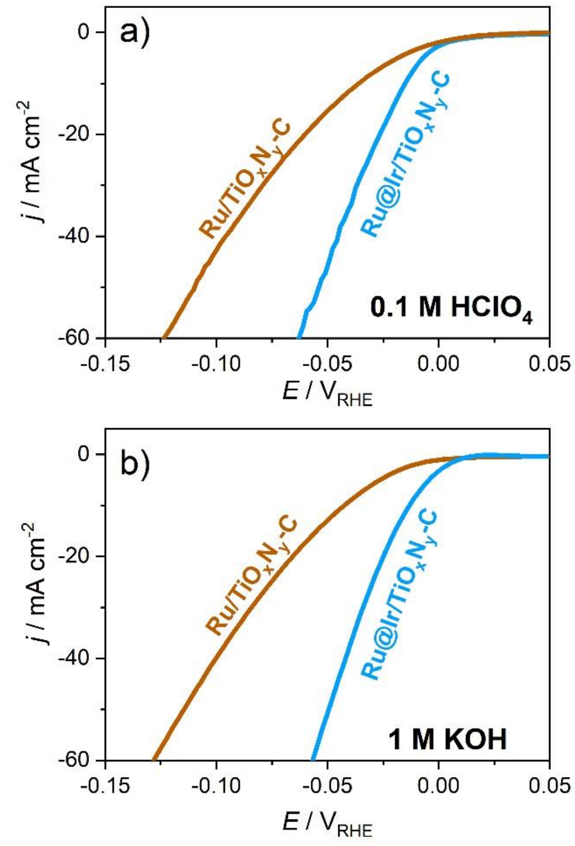

Figure S11. Comparison of HER activities of Ru/TiO_xN_y-C and Ru@Ir/TiO_xN_y-C samples in (a) acid and (b) alkaline electrolyte.

H adsorption to surface slabs

Table S1: Gibbs free energy for 1 H adsorption on Pt, Ir, Ru, and Ru@Ir surface slabs, as a function of the coverage of monolayer (ML) with adsorbed H (**θ**). All values are in eV.

**θ**	**0.0625 ML**	**0.125 ML**	**0.25 ML**	**0.5 ML**	**0.75 ML**	**1.0 ML**
**Ir**	–0.31	–0.30	–0.26	–0.25	–0.25	–0.24
**Ru**	–0.52	–0.52	–0.51	–0.48	–0.45	–0.42
**Ru@Ir**	–0.25	–0.25	–0.22	–0.22	–0.21	–0.21
**Pt**	–0.35	–0.34	–0.31	–0.27	–0.25	–0.24

Estimation of theoretical overpotential for HER

Theoretical overpotential for HER in acidic conditions U is estimated as:

$$U = \frac{|\Delta G_{\mathrm{H}}|}{e_0}$$

where e_0 is the elementary charge.

OH adsorption to surface slabs

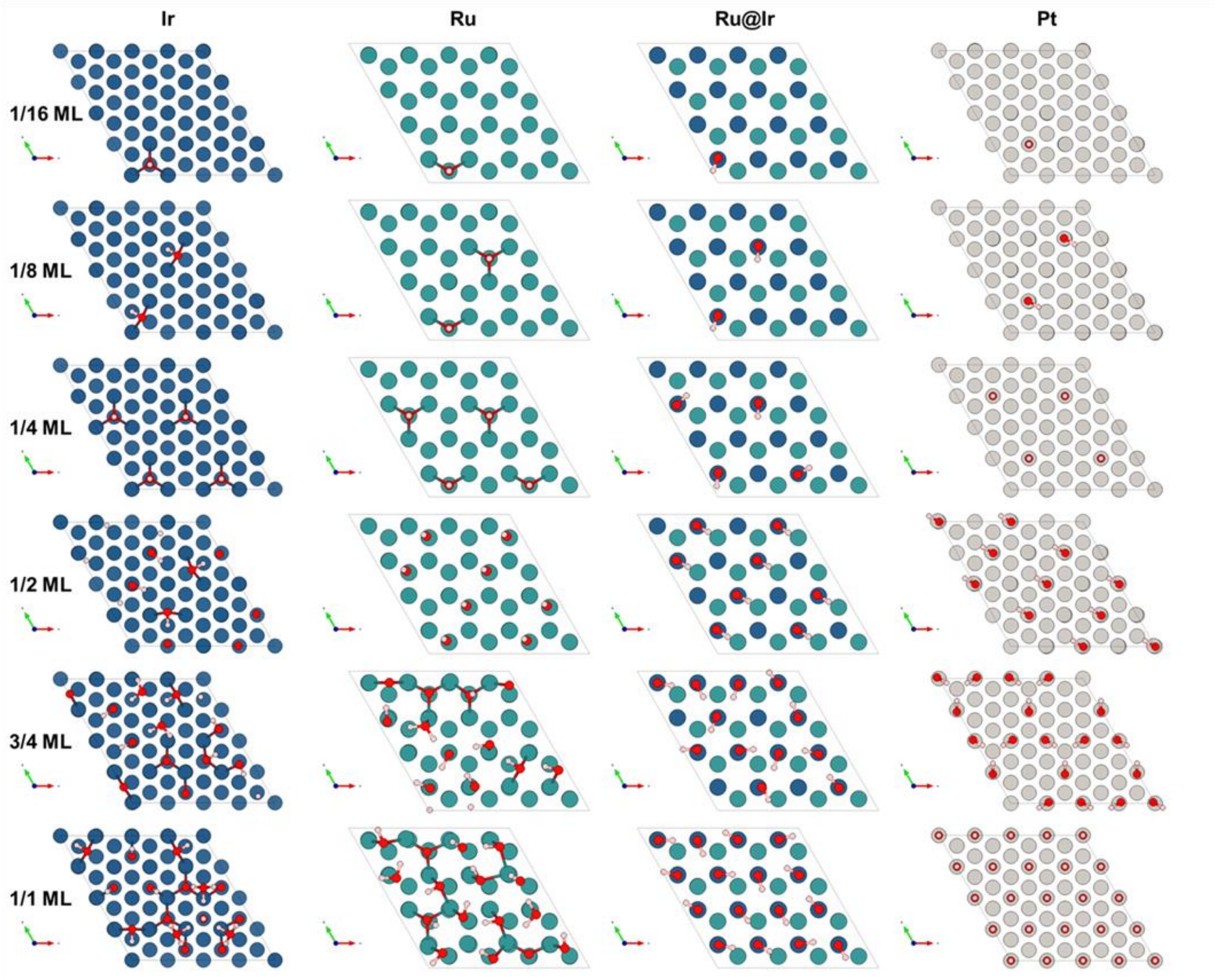

Figure S12. Configurations of adsorbed OH to Ir, Ru, Ru@Ir and Pt surface slabs as a function of surface coverage (ML = monolayer).

Table S2: Gibbs free energy for 1 OH adsorption on Pt, Ir, Ru, and Ru@Ir surface slabs, as a function of the coverage of monolayer (ML) with adsorbed OH (**θ**). All values are in eV.

θ	0.0625 ML	0.125 ML	0.25 ML	0.5 ML	0.75 ML	1.0 ML
**Ir**	0.81	0.59	0.90	0.43	0.65	0.78
**Ru**	–0.18	–0.12	0.01	0.28	–0.11	0.33
**Ru@Ir**	0.55	0.55	0.59	0.62	0.31	0.29
**Pt**	1.91	0.92	1.92	0.98	1.28	2.03

Differential charge density for Ru@Ir

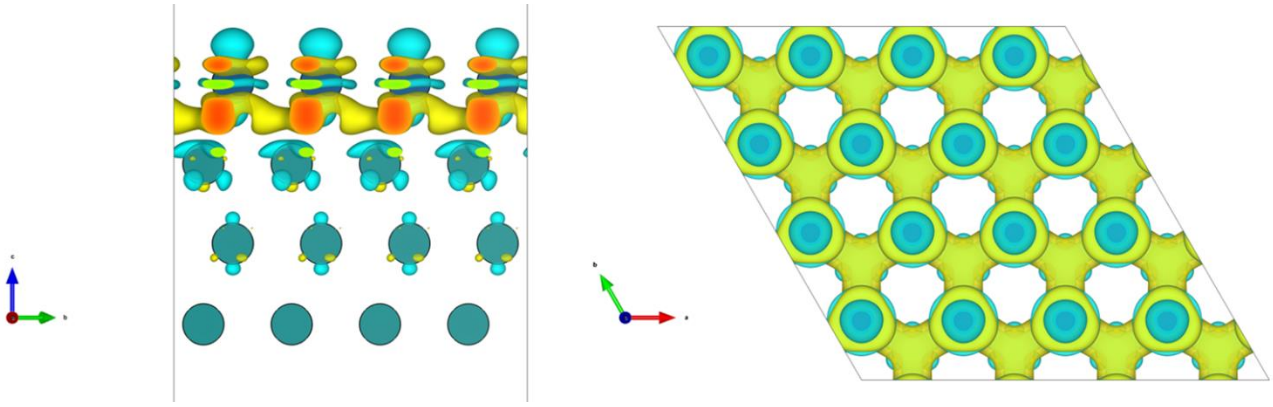

Figure S13. Differential charge density distribution for Ir@Ru surface slab.

Structure of titanium oxynitride surface slab

```
TiON slab
   1.0000000000000000
     5.8162660599000002    0.0000000000000000    0.0000000000000000
    -2.9081330299000001    5.0370341630000004    0.0000000000000000
     0.0000000000000000    0.0000000000000000   28.0596446991000015
   Ti   N    O
    9     8     8
Direct
  0.0067736364505819  0.0033868182252945  0.7015177864797479
  0.3265597067704107  0.6632798682281447  0.5100387673739206
  0.6666666866267761  0.3333333433259895  0.6057782769268343
  0.8370235318458867  0.6769795569156756  0.5155645769966810
  0.1666666716601242  0.3333333433259895  0.6057782769268343
  0.4963097816481294  0.9896871295377636  0.6959919768569875
  0.8370235318523171  0.1600440195612265  0.5155645769966810
  0.1666666716702352  0.8333333134472909  0.6057782769268343
  0.4963097816891633  0.5066226522010311  0.6959919768569875
  0.6703428350927894  0.8351713877612070  0.7282562365999468
  0.6629905378371106  0.8314952391333748  0.4833003172537289
  0.0011600896344319  0.5005800448959619  0.5648310732103425
  0.3321732536340249  0.1660866268662957  0.6467254806433260
  0.6574953533262686  0.3287476766757393  0.4872240613375922
  0.0000804953330586  0.0000402476665258  0.5656624569615332
  0.3332528478879340  0.6666264387869134  0.6458940968921354
  0.6758380199272835  0.3379190099762397  0.7243324925160834
  0.1845345655272084  0.8564595740429510  0.7289577587354259
  0.1487987778132620  0.8102070528516307  0.4825987951182498
  0.4976175230188034  0.4907800652864864  0.5661791944930883
  0.8357157905226771  0.1758866064757711  0.6453773593605803
  0.1845345655170973  0.3280749765873026  0.7289577587354259
  0.1487987778031510  0.3385917100646765  0.4825987951182498
  0.4976175229777695  0.0068374577819554  0.5661791944930883
  0.8357157905162467  0.6598292286714837  0.6453773593605803
```
